# Hydrogels and Dentin–Pulp Complex Regeneration: From the Benchtop to Clinical Translation

**DOI:** 10.3390/polym12122935

**Published:** 2020-12-09

**Authors:** Marwa M. S. Abbass, Aiah A. El-Rashidy, Khadiga M. Sadek, Sara El Moshy, Israa Ahmed Radwan, Dina Rady, Christof E. Dörfer, Karim M. Fawzy El-Sayed

**Affiliations:** 1 Oral Biology Department, Faculty of Dentistry, Cairo University, Cairo 11562, Egypt; marwa.magdy@dentistry.cu.edu.eg (M.M.S.A.); sarah.mahmoud@dentistry.cu.edu.eg (S.E.M.); esraa.ahmed@dentistry.cu.edu.eg (I.A.R.); dina.radi@dentistry.cu.edu.eg (D.R.); 2 Stem Cells and Tissue Engineering Research Group, Faculty of Dentistry, Cairo University, Cairo 11562, Egypt; aiah.abdelwahab@dentistry.cu.edu.eg (A.A.E.-R.); khadiga.sadek@dentistry.cu.edu.eg (K.M.S.); 3 Biomaterials Department, Faculty of Dentistry, Cairo University, Cairo 11562, Egypt; 4 Clinic for Conservative Dentistry and Periodontology, School of Dental Medicine, Christian Albrechts University, 24105 Kiel, Germany; doerfer@konspar.uni-kiel.de; 5 Oral Medicine and Periodontology Department, Faculty of Dentistry, Cairo University, Cairo 11562, Egypt

**Keywords:** hydrogels, polymers, stem cells, tissue engineering, dental, regeneration

## Abstract

Dentin–pulp complex is a term which refers to the dental pulp (DP) surrounded by dentin along its peripheries. Dentin and dental pulp are highly specialized tissues, which can be affected by various insults, primarily by dental caries. Regeneration of the dentin–pulp complex is of paramount importance to regain tooth vitality. The regenerative endodontic procedure (REP) is a relatively current approach, which aims to regenerate the dentin–pulp complex through stimulating the differentiation of resident or transplanted stem/progenitor cells. Hydrogel-based scaffolds are a unique category of three dimensional polymeric networks with high water content. They are hydrophilic, biocompatible, with tunable degradation patterns and mechanical properties, in addition to the ability to be loaded with various bioactive molecules. Furthermore, hydrogels have a considerable degree of flexibility and elasticity, mimicking the cell extracellular matrix (ECM), particularly that of the DP. The current review presents how for dentin–pulp complex regeneration, the application of injectable hydrogels combined with stem/progenitor cells could represent a promising approach. According to the source of the polymeric chain forming the hydrogel, they can be classified into natural, synthetic or hybrid hydrogels, combining natural and synthetic ones. Natural polymers are bioactive, highly biocompatible, and biodegradable by naturally occurring enzymes or via hydrolysis. On the other hand, synthetic polymers offer tunable mechanical properties, thermostability and durability as compared to natural hydrogels. Hybrid hydrogels combine the benefits of synthetic and natural polymers. Hydrogels can be biofunctionalized with cell-binding sequences as arginine–glycine–aspartic acid (RGD), can be used for local delivery of bioactive molecules and cellularized with stem cells for dentin–pulp regeneration. Formulating a hydrogel scaffold material fulfilling the required criteria in regenerative endodontics is still an area of active research, which shows promising potential for replacing conventional endodontic treatments in the near future.

## 1. Introduction

Regeneration of damaged/lost biological tissues represents a major challenge for clinicians and researchers worldwide. The extracellular matrix (ECM) of these biological tissues is composed of a tangled mesh of fibrous proteins and glycosaminoglycans, which provides anchorage to the cells and regulates cellular behavior and interaction via various signals. ECM is synthesized and continuously remodeled by its resident cells [1]. Natural and synthetic biomaterials that can mimic ECM to reproduce all its characteristics are still not available [2]. However, currently the use of natural scaffolds, including polymeric scaffolds, ceramic scaffolds or combinations of different scaffold types loaded with suitable cells and signaling molecules is considered the classical strategy in tissue engineering approaches.

Dentin–pulp complex is a term referring to the dental pulp surrounded by dentin along its peripheries. It reflects the close anatomical and functional relationship that exists between the dentin and dental pulp [3]. Dentin and dental pulp are highly specialized tissues, where the dental pulp is a vascular connective tissue responsible for the maintenance of tooth vitality, while dentin is the protective tissue for this vital pulp [4,5,6]. Maintaining dentin/pulp integrity and vitality are of importance for all dental practitioners and researchers [7]. Dental caries, among other insults to the tooth structure, can result in irreversible pulpal damage with devastating effects. Regeneration of the dentin–pulp complex to regain tooth vitality remains to be of paramount importance.

Dentin is composed of an inorganic (hydroxyapatite) and organic matrix, comprising mainly collagen and non-collagenous proteins. Non-collagenous proteins include phosphorylated proteins, members of the small integrin-binding ligand N-linked glycoproteins (SIBLINGs) family, including transient dentin sialophosphoprotein (DSPP), which is cleaved after secretion into dentin sialoprotein (DSP) and dentin phosphoprotein (DPP), in addition to dentin matrix protein-1 (DMP-1), bone sialoprotein (BSP) and osteopontin (OP). Non-collagenous proteins also include non-phosphorylated proteins, including osteocalcin (OCN) and small leucine-rich proteoglycans. Non-collagenous proteins have cell-binding sequences, arginine–glycine–aspartic acid (RGD) domains, and can act as signaling molecules in addition to their role in controlling dentin mineralization and regulating hydroxyapatite crystal formation [8]. Three types of dentin can be distinguished: primary dentin, that is formed before tooth eruption, secondary dentin, formed after completion of root formation and tertiary dentin, formed in response to various stimuli, reflecting the dentin–pulp complex’s regenerative capacity. According to the urgency of the stimulus, tertiary dentin can be classified into reactionary dentin formed by existing odontoblasts in response to mild stimulus and reparative dentin formed by odontoblasts-like cells recruited from pulp progenitor cells in response to more severe stimuli [9].

The dental pulp, on the other hand, is a loose connective tissue occupying the center of the tooth. It is a home to a variety of cells, including fibroblasts, odontoblasts, inflammatory and immune system cells in addition to dental pulp stem cells (DPSCs), which reside in perivascular areas and the cell-rich zone of Hohl [9,10]. Dental pulp is also characterized by its extensive neuro-vascular networks enabling it to perform its functions, which should be duplicated for successful pulpal regeneration [11].

Odontoblasts are specialized cells responsible for the formation of dentin. They originate through the differentiation of ecto-mesenchymal cells of the dental papilla triggered by a series of complex epithelial mesenchymal interactions [3,12]. The process of odontoblastic differentiation is closely governed by bioactive molecules, which can promote cell proliferation, migration and differentiation. Various growth factors, including transforming growth factor-beta (TGF-β), bone morphogenic proteins (BMP), epidermal growth factor, fibroblast growth factor 2 (FGF2), growth differentiation factor 11, platelet-derived growth factor (PDGF), hepatocyte growth factor and insulin-like growth factors (IGF), have been implicated in odontoblastic differentiation during early stages of tooth development [3]. Once differentiated, odontoblasts can in turn secrete bioactive molecules including TGF-β, BMPs, insulin-like growth factor (IGF-1) and IGF-2, FGF-2 and angiogenic factors. Those bioactive molecules become entrapped within the dentin matrix and can be released upon matrix degradation into the pulp tissues to undertake a role in the regeneration process [9,13]. Odontoblasts’ response to various stimuli, such as dental caries or cavity preparation, is governed by the intensity of the inflammatory reaction. Low-grade inflammation or stimulus induce dentin regeneration through bioactive molecules released from the dentin matrix and pulpal cells, while severe stimuli and inflammation induce cellular death and impair dentin regeneration [14].

For damaged pulp tissue, direct and indirect pulp capping are considered to be the first line of treatment to maintain the pulpal tissue vitality, while the endodontic treatment, relying on three-dimensional shaping, cleaning and filling of the pulpal soft tissue space within the tooth via a biocompatible inert material, leads to loss of pulp vitality with its consequences on the integrity of the tooth structure. In young permanent teeth, such a loss of vitality may affect the tooth maturation and its apexogenesis, which renders the regeneration of the dentin–pulp complex through a different tissue engineering approach a major concern for researchers. On the other hand, dentin–pulp complex regeneration through regenerative endodontic procedure (REP) [15,16] or revascularization relies on stimulating the differentiation of resident stem/progenitor cells [17]. REP involves the induction of intracanal bleeding and the formation of a blood clot, which act as a scaffold for stem/progenitor cells from the apical dental papilla (SCAP) migration and differentiation for regeneration [18].

For successful REP, two prerequisites are needed to achieve pulp regeneration; efficient root canal disinfection and proper size of the apical foramen [19]. Thus, the effect of different root canal irrigants used on cellular behavior and differentiation is crucial. Sodium hypochlorite (NaOCl) has a potent anti-microbial and proteolytic activity, however, it could cause a severe inflammatory response and a damage to vital tissues. Furthermore, NaOCl cannot remove the smear layer on the dentin surface, which may make the dentin surface unrecognizable to the seeded cells and hinder cellular interaction [20]. Ethylenediaminetetraacetic acid (EDTA) on the other hand has a chelating activity and is used as a final rinse for smear layer removal, or in combination with NaOCl and chlorhexidine gluconate (CHX) solutions. The addition of EDTA to other rinsing solutions can increase the viability of DPSCs, and induce DPSCs cell attachment and odontoblastic/osteoblastic differentiation [21]. Additionally, EDTA can remove the smear layer and stimulate the release of pro-angiogenic GFs in the dentin matrix, including TGF-β, VEGF, FGF-2, PDGF, and BMP-2, through the demineralization of the superficial dentin layer [20,22].

Dentin–pulp complex regeneration could also include approaches for replacing/repairing the damaged pulp tissues through tissue engineering approaches. Different tissue engineering approaches depend basically on the combination of three components: cells, bioactive molecules and scaffolds. Stem/progenitor cells investigated in the field of dentin–pulp complex regeneration include stem/progenitor cells of exfoliated deciduous teeth (SHED), periodontal ligament stem/progenitor cells (PDLSCs), DPSCs, SCAP, and dental follicle stem/progenitor cells [23]. BMP, vascular endothelial growth factor (VEGF), FGF-2 and TGF are the principal morphogens used frequently in conjunction with dental stem/progenitor cells to induce a variety of cellular activities and induce various tissue structures, even when used at very low concentrations [1]. VEGF and FGF were shown to enhance angiogenesis and neovascularization in severed human dental pulps [24], while BMPs are suggested to induce new dentin formation [25]. Scaffolds used for dentin–pulp complex regeneration include polymeric, ceramics and bioactive glass [7,11], whereby the scaffold carries bioactive molecules that can home, stimulate and promote differentiation of tissue resident stem/progenitor cells [26]. Cell-free scaffolds were further suggested as an alternative for dentin–pulp complex regeneration.

**Figure 1:**
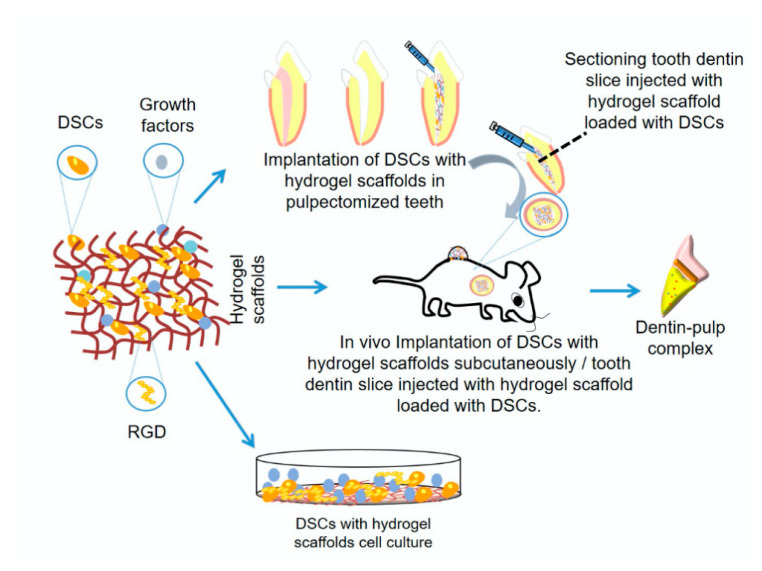
Schematic diagram showing hydrogel scaffold for dentin/pulp regeneration research methods.

Recently, hydrogel-based scaffolds were introduced in the field of tissue engineering. They are a unique category of three-dimensional (3D) polymeric networks with water as the liquid component. Their hydrophilic nature renders them able to retain high water content and biological fluids, as well as diffusion of nutrients through their structure. In addition to their biocompatibility, their expected degradation pattern and their adjustable mechanical properties, they can maintain their network integrity and thus do not dissolve in high water concentrations due to their crosslinking structure. Furthermore, hydrogels have a considerable degree of flexibility and elasticity similar to a natural ECM, providing the essential cell support needed during tissue regeneration. Thus, they are considered an optimal choice for many tissue engineering applications due to such unique characteristics, in addition to their gelatinous structure and their ability to be loaded with different drugs, making them successful drug delivery system [7,27,28,29] (Figure 1).

Hydrogel systems can be classified according to their polymeric composition, origin, physical appearance, configuration, type of crosslinking or electrical charge [30]. According to the source of the polymeric chain forming the hydrogel, they can be classified into natural, synthetic or hybrid hydrogels. Hydrogels could be made essentially from one type of polymer, natural or synthetic, or a combination of them, or by modifying the polymer through crosslinking agents. The natural polymers forming hydrogels are mainly collagen, fibrin, chitosan, alginate or hyaluronic acid, while the synthetic ones are the polylactic acid (PLA)-based hydrogels, polydimethylsiloxane, polyethylene glycol (PEG), or self-assembling peptides. However, although natural polymers-based hydrogels are characterized by their ability to mimic natural tissues, they are also liable to be easily permanently damaged due to their poor mechanical properties. On the contrary, the synthetic ones have considerably higher mechanical properties and tunable physicochemical properties but lack natural tissue resemblance [29].

Depending on the crosslinking method, hydrogels are either physically or chemically crosslinked [31,32]. Crosslinking is essential for hydrogel solidification to attain adequate mechanical properties, prevent its premature dissolution in an aqueous environment, increase its resistance to heat or wear and to create a network structure [33,34]. Physically crosslinked hydrogels are formed by self-assembly following a change in environmental conditions, including temperature, ionic concentration or pH value [35]. Physically crosslinked hydrogels are stabilized via physical interaction between polymeric chains, without the use of any chemical crosslinkers or initiators, while chemically crosslinked hydrogels are stabilized by covalent bonding between polymeric chains [33].

## 2. Requirements of Ideal Hydrogel Scaffold for Dentin–Pulp Complex Regeneration

**Figure 2:**
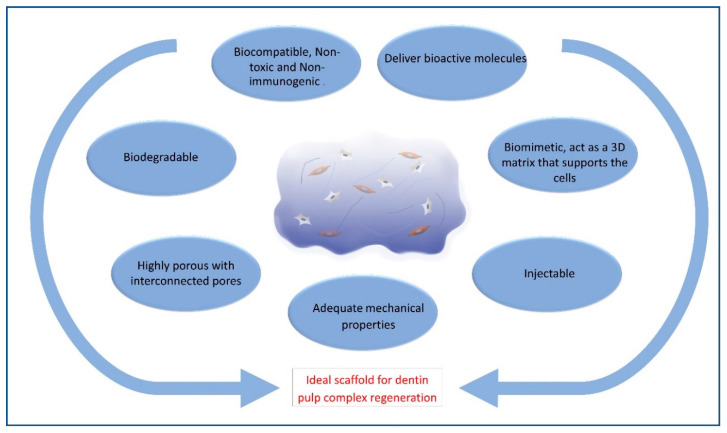
Schematic diagram showing criteria of ideal hydrogel scaffold.

The ideal materials used for dentin–pulp complex regeneration (Figure 2) should be biocompatible and clinically applicable. They should be sterilizable, easy to use and apply, have the ability to be stored at clinical settings, with a reasonable shelf-life, injectable to adapt to canal morphology, have short setting times and be without any discomfort to the patient [36,37]. Scaffolds should also be cost-effective to allow mass production for clinical translation [38].

On the biological level, hydrogel scaffolds used for dentin–pulp complex regeneration and their degradation by-products should be biocompatible, non-toxic, non-immunogenic, and should not induce significant inflammatory reactions. They also should allow the encapsulation or surface adhesion of cells, and promote cellular migration, proliferation, differentiation and function [37,39]. Ideally, the hydrogel should solidify at neutral pH and at physiological temperature to avoid cellular damage. Changes in temperature, pH or free radicals released during gelation can negatively affect loaded bioactive molecules or cell viability [40]. Natural hydrogels carry a lower risk for cytotoxicity [41]. Glutaraldehyde crosslinked gelatin hydrogels showed significantly lower biocompatibility as compared to 1-ethyl-3-(3-dimethyl aminopropyl) carbodiimide crosslinking in vitro and in vivo [41]. Synthetic hydrogels, on the other hand, carry a higher risk of cytotoxicity. It is noteworthy that hydrogels are injected before the gelation process, which imposes a higher risk for cytotoxicity. Therefore, all hydrogel components should be thoroughly examined for their cytotoxic effects. The small molecules used for the gelation process, such as unreacted monomer, initiator and crosslinkers, that can elicit cellular toxicity, should be carefully removed for safe clinical application [7,33]. Additionally, hydrogels derived from animal sources could be immunogenic [7].

The hydrogel scaffold should be degradable upon implantation to be replaced by newly formed tissues [42]. Ideally, the rate of scaffold degradation should be compatible with the rate of new tissue formation. Too-rapid scaffold degradation can compromise its cell-supporting function, while a too-slow degradation rate can hinder new tissue formation [26,43]. Hydrogels may undergo degradation via simple dissolution, hydrolysis, enzymatic degradation or a combination of the previous mechanisms. Simple dissolution is common with physically crosslinked hydrogels, whereby the hydrogel does not break into smaller molecules but rather dissolves in a solution with change in environmental conditions [7]. Hydrolysis, which is the most common mechanism for hydrogel degradation [44], occurs hereby via the breakdown of labile ester linkages in the polymer chain, while enzymatic degradation occurs via enzymatic cleavage of peptide bonds incorporated into the hydrogel polymeric chains [45,46].

Scaffolds should have high porosity and interconnected pores, to allow vascularization, nutrients and waste diffusion, as well as cellular migration [7,47]. Increasing internal pores can compromise mechanical properties, while increasing gelatin pores can reduce material stiffness [48]. Adequate mechanical properties are essential for hydrogel scaffolds to provide support for the cells and withstand mechanical loading [47,49]. Hydrogel mechanical properties largely depend on hydrogel composition, concentration, method of fabrication in addition to crosslinking density, porosity and hydrogel modification [50,51,52]. Increasing the material mechanical properties can be achieved by increasing crosslinking density and decreasing its porosity, which can compromise degradability and cellular response [7,47]. Therefore, a balance should exist between hydrogel mechanical properties and degradability [33].

For dentin–pulp complex regeneration, injectable hydrogels combined with stem/progenitor cells is considered a promising approach in the tissue engineering field. This is primarily attributed to their ability to be injected inside the tooth and to adapt to the contour of the pulp chamber easily and successfully. Their gelation in situ occurs by the crosslinking of hydrogel precursors, making them able to fill all irregularities and all defects properly [53]. The material should be flowable at the time of injection and solidify once in place in response to changes in temperature, pH, light, enzyme or the addition of a crosslinking agent [7]. Furthermore, the scaffold material should be biomimetic and act as a 3D matrix that supports the cells and mimics the ECM. It should also provide bioactive molecules, and promote cells attachment, proliferation, survival and differentiation to optimize tissue regeneration [47,54,55]. The hydrogels can further be biofunctionalized to enhance cellular response through incorporation of cell-binding sequences such as RGD into the hydrogel [56]. Further, hydrogels can be used for the local delivery of bioactive molecules, such as TGF- ꞵ1, BMP-2, VEGF or PDGF, which might consequently support odontogenic differentiation [57]. These bioactive molecules can be released by diffusion, degradation, cleavage of the hydrogel or combination of the previous mechanisms [33]. Hydrogel drug release kinetics involve an initial burst release followed by a diffusion-dominated release phase, then a stage of combined diffusion and degradation, and finally the carried drug is released through polymer degradation [58].

## 3. Mechanism of Action of Hydrogels in Dentin–Pulp Regeneration 

**Figure 3:**
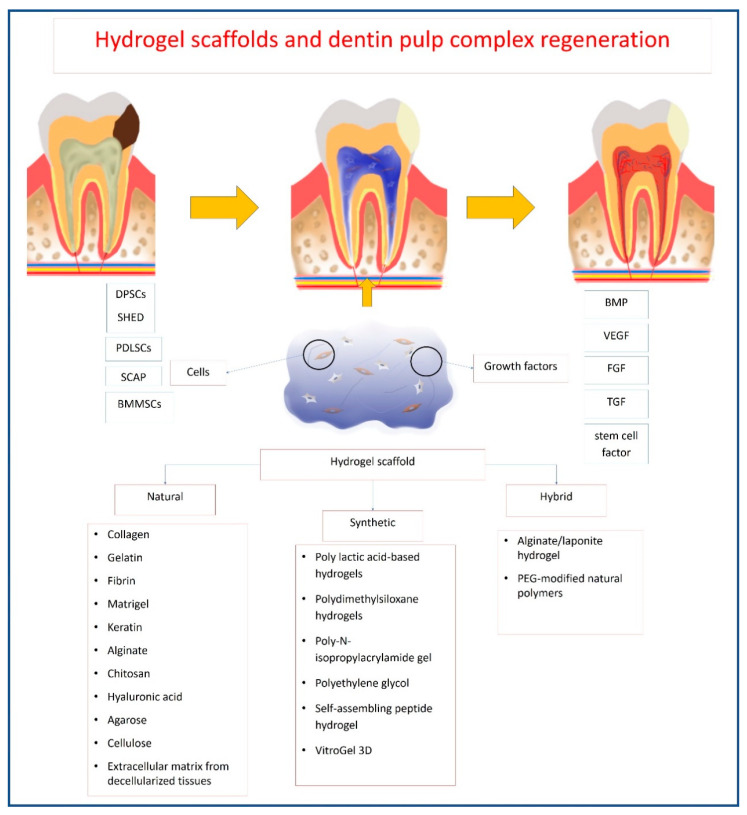
Schematic diagram showing mechanism of hydrogel action for dentin–pulp complex regeneration.

The hydrogels’ mechanisms of action in vivo include their role as a space filling material, in addition to their roles as carriers for cells and bioactive molecules [59]. Hydrogels should maintain the desired volume and structural integrity for the required time to perform this function [59]. For dentin–pulp complex regeneration, hydrogels act as carriers of stem/progenitor cells with odontogenic potential, such as DPSCs [60,61,62,63,64,65,66,67], odontoblasts-like cells [68,69,70], HUVECs [64,71], SCAP [72,73], SHED [66,74,75,76], BMMSCs [66,77,78,79,80], PDLSCs [66], endothelial cells [78] and primary dental pulp cells [81]. They can also act as carriers for the local delivery of antibiotics, such as clindamycin [82] and bioactive molecules, aiming to promote tissue regeneration, such as VEGF [20,61,83], FGF [20,83,84,85], BMP [65], TGF-β1 [20,86], stem cell factor [87], dentonin sequence [88] and RGD cell-binding motifs [56,89]. Once implanted in site, being biodegradable, hydrogels allow the release of bioactive molecules that influence the surrounding environment [90,91] (Figure 3).

In this review, we aim to discuss the different hydrogels used in dentin–pulp complex regeneration, combined or not with stem/progenitor cells. As the polymer is the backbone forming any hydrogel, the classification of hydrogels in this review is based upon the nature of the main polymer/polymers forming the hydrogel.

## 4. Hydrogels Used in Dentin–Pulp Complex Regeneration

### 4.1. Natural Hydrogels

Naturally derived polymers are frequently used as injectable biomimetic hydrogel materials owing to their bioactivity and ability to interact with cells, as well as their biocompatibility and biodegradability with naturally occurring enzymes or through hydrolysis [92]. Natural polymers are either natural polypeptides of the ECM (e.g., collagen, fibrin, gelatin and keratin) or chemically similar to natural glycosaminoglycans (e.g., alginate, chitosan and hyaluronic acid). Most of the natural polymers are bioactive, containing cellular binding motifs, thus promoting cell adhesion, and/or present soluble signaling factors capable of regulating cell behavior. However, natural polymers suffer from certain limitations, including batch-to-batch variations, poor mechanical properties and uncontrolled degradation rate, compromising the materials’ chemical and physical properties over time. Moreover, natural hydrogels may be inherently immunogenic or give rise to some immunogenicity due to the presence of contaminants, like proteins and endotoxins [93,94], in addition to the potential risk of transmitting pathogens from animal sources [92].

#### 4.1.1. Collagen

Collagen is an insoluble fibrous protein of vertebrates that is the chief constituent of the fibrils of the connective tissue of skin, tendon and bone. The presence of collagen in all connective tissues makes it one of the most studied biomolecules of the ECM [95]. Collagen has a structural and chemical similarity to the predominant structural protein found in the ECM of several dental tissues. Collagen is highly biocompatible and bioactive, promoting cellular adhesion, cellular migration and cell proliferation. It is enzymatically biodegradable by collagenase. Although collagen has poor mechanical properties with high tensile strength, it is considered sufficient for pulpal tissue regeneration. The crosslinking of collagen can modify its mechanical and physical properties, however the added chemical agents could compromise cell survival and biocompatibility [96].

Crosslinking collagen hydrogel with cinnamaldehyde (CA), the most important constituent of cinnamon, significantly shortened the setting time, and increased compressive strength and surface roughness. CA per se did not increase alkaline phosphatase (ALP) activity, calcium nodule formation or the expression of odontogenic-related markers of human DPSCs cultured in a collagen hydrogel. The odontogenic markers investigated included DSPP, DMP-1, matrix extracellular phosphoglycoprotein (MEPE) and osteonectin (ON), while dental pulp cell proliferation and odontogenic differentiation cultured in collagen scaffolds were promoted in the presence of CA, as evident by the significantly higher expression of DSPP, DMP-1 and MEPE. Dental pulp cells spread across the CA crosslinked collagen, showing numerous cytoplasmic extensions, unlike cells in the uncrosslinked collagen matrix. This could be attributed to the enhanced physical properties of collagen by CA-induced crosslinking that upregulated the cellular adhesion, as compared to the sol state [60]. Variations in mechanical properties, such as stiffness, were shown to modulate human embryonic stem cells (ECs) differentiation. Substrate stiffness could act as a biomechanical regulatory factor through constraining the movement of tissues and cells. On stiffer matrices, cell movements becomes slower, cells exert higher tractions forces on their substrates and cell-generated forces are dissipated within the cell, possibly changing the protein conformation connecting the cytoskeleton and matrix [97,98]. This could explain the impact of increased compressive strength on human DPSCs count and odontogenic differentiation [60].

Thus, collagen matrices with tunable mechanical properties (e.g., stiffness) could affect cell–matrix interactions. Human amniotic membrane, which is composed of a single epithelial cell layer supported by a collagen-rich layer, is commonly used in ocular regeneration [99]. Crosslinked human amniotic membrane demonstrated higher rigidity and roughness, and positively influenced human limbal epithelial cells in vitro via the Wnt/β catenin pathway as compared to non-crosslinked amniotic membrane [100].

The stiffness and microstructure of collagen matrices could be tailored by modulating collagen concentration and the oligomer:monomer ratio [101]. Collagen hydrogels with varying stiffnesses were prepared by varying oligomer concentrations of 1.37 mg/mL (235 Pa) for endothelial differentiation, and 2.88 mg/mL (800 Pa) for odontogenic differentiation. The effects of the incorporation of VEGF into 235 Pa oligomeric collagen, or BMP-2 into the 800 Pa oligomeric collagen, on DPSC’s viability and differentiation were also investigated. DPSCs-laden oligomeric collagen gels with stiffnesses of 235 and 800 Pa demonstrated the slow release of simulated growth factors (GFs), supported long-term cell survival and favored the differentiation of cells to a specific lineage. The effect of stiffness on the cytoskeletal organization and cell shape was further evident, whereby stiffer collagen (800 Pa) decreased the cell spreading and actin fiber organization, while softer collagen supported the growth of elongated DPSCs [61]. Real-time polymerase chain reaction (RT-PCR) data showed that the DPSCs cultured in 235 Pa matrices demonstrated an increased expression of endothelial markers, Von Willebrand Factor, platelet endothelial cell adhesion molecule-1 (PECAM-1) and vascular endothelial-cadherin after 28 days. The effect seemed to be enhanced upon VEGF incorporation. The 800 Pa DPSCs-laden oligomeric collagen matrices induced osteogenic differentiation, as was evident by an increase in ALP activity, and higher mineralization evident by Alizarin S staining demonstrated on day 21, an effect further amplified by BMP-2 incorporation [61].

Culturing DPSCs with their natural associated dental pulp ECM (pECM) was subsequently suggested to better mimic the cells’ natural environment for enhanced cell culture and differentiation [81]. pECM is a loose highly hydrated viscous extracellular connective tissue matrix of collagenous and non-collagenous proteins, rich in hyaluronan, glycosaminoglycans and proteoglycans, all held together in a network of thin collagen fibrils, reticular fibrils and fibronectin. Col I and III are the two major structural components of the pECM [102]. The non-collagenous component contains a significant number of well-characterized bioactive regulatory molecules, such as DSP, DPP, BSP, DMP-1, OP, MEPE and members of SIBLINGs [103], in addition to other various non-phosphorylated proteins, various GFs and their receptors, and a variety of enzymes [102]. Cells grown on bovine pECM-coated cultureware did not demonstrate enhanced proliferation as compared to cells grown on uncoated control. On the contrary, the cells showed an abundant expression of stem/progenitor cells markers (including CD44, VIM and Sox2) and higher levels of pluripotent transcription factor Oct ¾, as compared to controls. Cells grown on uncoated control showed higher expression of the odontogenic cell fate markers (DSPP and DMP). The data suggested that cells cultured on pECM maintained undifferentiated stem/progenitor cell phenotype, compared to their controls [81].

To avoid the risk of ectopic mineralization, determining the fate of implanted cells in the pulp is an important consideration. Type I collagen hydrogel was employed to track the fate of rat pulp cells labeled with indium-111-oxine (111In-oxine), implanted in the empty pulp chamber space of a rat’s upper first molar, by helical single-photon emission computed tomography (SPECT)/computed tomography (CT). One month following implantation, active fibroblasts, new blood vessels and nerve fibers were present in the cellularized 3D collagen hydrogel. SPECT provided a non-invasive follow-up of the implanted cells for up to 3 weeks, wherein signal intensity reflected implanted cell integrity. Reconstructed SPECT and CT scans revealed retention of the implanted cells in the pulp space with no evidence of systemic release [104].

#### 4.1.2. Gelatin Hydrogel

Gelatin is a natural collagen derivative acquired through the hydrolysis of collagen triple helix into single molecules via alkaline or acid treatment processes [105]. Furthermore, gelatin is a biopolymer able to exhibit a thermoreversible sol–gel transition, whereby at higher temperatures it melts, and returns to a gel at lower temperature. This allows for fabricating cell/tissue encapsulation carriers using gelatin [106]. Gelatin is biocompatible, being natural, hydrophilic, biodegradable and non-immunogenic as compared to collagen [107,108]. The bloom value of gelatin (a method of measuring gel strength) can influence its properties. Gelatin with low bloom value has a low melting point, and a higher biocompatibility, which further endorse its use as a cell carrier for tissue regenerative purposes [106].

Gelatin retains RGD cell-binding motifs, which promote cellular attachment [109], as well as matrix metalloproteinase (MMP) binding domain, which allows its biodegradation [110]. Gelatin can also promote cellular proliferation and differentiation [111,112] as well as DPSCs adhesion, proliferation, migration and odontogenic differentiation, as was evident by an increased mineralization, ALP activity and an increased expression of collagen I (Col I), OCN, DSPP and DMP-1 [108]. Gelatin is relatively inexpensive and readily available [113]. However, it has poor mechanical properties [114,115] and is thermally unstable, as it is converted into a sol at temperatures above 37 ℃ [116]. These shortcomings can be overcome by gelatin crosslinking, which involves adding chemical groups to gelatin active side chains, including –OH, –COOH, –NH2 and –SH groups [116].

In order to improve its biological properties, gelatin hydrogel was used for the local delivery of FGF [84,85,117] and simvastatin for dental tissue regeneration [63]. Crosslinked gelatin hydrogel microspheres were impregnated with FGF-2 and mixed with pieces of gelatin sponge before implantation in rats’ exposed dental pulp. Dentin particles were formed within the dentin defect and dental pulp cells showed an increased expression of DSPP [84]. Similarly, gelatin hydrogel microspheres incorporating different dosages of FGF-2 were associated with dentin regeneration. The quality of regenerated dentin was dependent on the concentration of FGF-2. FGF-2 moderated a dose-induced formation of dentin bridge upon implantation in rats’ molars, and was associated with increased DMP–1 expression [117].

Furthermore, simvastatin–lactic acid-grafted gelatin micelles were fabricated through the incorporation of simvastatin into gelatin laden with lactic acid oligomer, which were further mixed with gelatin, followed by chemical crosslinking to form biodegradable gelatin hydrogels. Simvastatin-releasing hydrogel effectively increased ALP expression in DPSCs in vitro. It was also associated with increased calcification and DSP expression in vivo upon ectopic implantation in immunocompromised mice. The enhanced DPSCs’ biological activity was attributed to increased levels of BMP-2 expression [63].

Gelatin hydrogels were enzymatically crosslinked with microbial transglutaminase to create a scaffold with variable stiffness. Crosslinked gelatin hydrogel substrates were associated with increased mineralization, odontogenic differentiation of DPSCs and increased expression of odontoblasts’ markers, including OCN, DSPP and ALP, regardless of matrix stiffness or chemical stimulation using dexamethasone in vitro. The stiffness of the gelatin matrix was associated with the permanent differentiation of DPSCs even after being removed from the gelatin hydrogel surface [62].

Gelatin methacrylate (GelMA) is a gelatin-based hydrogel [118] derived through the reaction of gelatin with methacrylic anhydride, replacing amino groups present on the side chains of gelatin with methacryloyl groups. GelMA usually polymerizes under ultraviolet light, in the presence of a photo-initiator, into thermostable crosslinked hydrogel [116,119]. Being derived from gelatin, GelMA is biocompatible, and retains RGD cell-binding motifs and the MMP-binding domain, which support cell attachment and GelMA biodegradation, respectively [116]. GelMA can support cellular adhesion, proliferation, migration and organization, besides being relatively inexpensive [120]. GelMA is characterized by being thermally stable and can retain its physical properties at body temperature [121]. Additionally, GelMA is an ideal candidate for dentin–pulp complex regeneration as it can be easily injected into the exposed pulp cavity and photo-polymerized [64]. A GelMA hydrogel loaded with cells can be photopolymerized using dental curing light to facilitate its clinical translation for dental regeneration [122]. It has poor mechanical properties; however, it can be modified to increase its mechanical properties [118] through controlling the degree of methacrylation [120] or the polymer concentration [72].

Matrix stiffness and micropatterning of GelMA hydrogel scaffold can be altered to resemble odontoblasts’ native environment, thus enhancing cellular response and behavior [68,72]. Increasing GelMA hydrogel stiffness through increasing the polymer concentration significantly increased SCAP proliferation [72] and odontoblasts-like cells’ proliferation, viability and migration in vitro [68]. Additionally, micropatterning of the GelMA hydrogel via photolithograph promoted SCAP alignment and odontogenic differentiation [72]. Pre-vascularized hydrogel scaffolds with microchannels were fabricated through injecting GelMA hydrogel loaded with odontoblasts-like cells into the root fragment followed by photopolymerization. Odontoblasts-like cells encapsulated within GelMA hydrogel showed a tendency to spread close to dentinal walls, while endothelial colony-forming cells seeded in the microchannels formed endothelial monolayers and angiogenic sprouting in vitro [68].

In vivo findings also confirmed the suitability of cell-loaded GelMA hydrogel for dentin–pulp complex regeneration. Human DPSCs and human umbilical vein endothelial cells (HUVECs) suspended in GelMA hydrogel were injected into human root segments, sealed at one end using mineral trioxide aggregate. This was followed by the photo crosslinking of cells loaded with GelMA hydrogel. Root segments were then implanted subcutaneously in rats. Highly cellular and vascular pulp-like tissue was formed in the root segments. Further, GelMA remnants and cells became attached to the dentinal walls, and DPSCs developed cellular extensions into the dentinal tubules and secreted a matrix resembling reparative dentin [64].

Aiming to accelerate the formation of the intracanal blood clot required for dental pulp regeneration and to control pulpal bleeding, thrombin solution, a hemostatic solution, was added to the GelMA hydrogel before loading with DPSCs. An access cavity was prepared in the immature premolars of minipigs, the pulp was extirpated and the root canals were mechanically prepared. This was followed by over-instrumentation, followed by injection of the hydrogel loaded with cells into the canal, and tooth restoration. Cell-loaded GelMA was biocompatible as the DPSC’s vitality was not affected, and an inflammatory response was not elicited in vitro. GelMA was also associated with successful apex maturation and the formation of vascular pulp-like tissue. Additionally, odontoblasts lining the pulp cavity and deposition of reparative dentin along dentinal walls were detectable. DPSCs-laden GelMA showed more promising results as compared to DPSCs-laden fibrin hydrogel, which was associated with periapical radiolucency and internal root resorption [67].

GelMA can be modified for the local delivery of growth factors. GelMAs conjugated with synthetic BMP-2 mimetic peptide and loaded with human DPSCs were prepared in bioink for the 3D bioprinting of stem cells-loaded dental constructs. DPSCs maintained viability, and displayed increased proliferation, mineralization and osteogenic differentiation, in addition to the expression of DSPP and OCN in vitro [65].

#### 4.1.3. Fibrin

Fibrin is a naturally derived insoluble protein biopolymer, produced through the polymerization of fibrinogen protein (present in blood plasma) under the control of thrombin during blood clotting, which results in the formation of a fibrous polymer network important in hemostasis and wound healing [123]. Fibrin offers several advantages as compared to synthetic polymers and collagen in being of low cost, providing excellent biocompatibility and superior cell adhesion properties. It can be easily obtained from autologous sources, thus avoiding undesirable immunogenic reactions [96]. Fibrin is biodegradable in a controllable manner, has non-toxic degradation products and can be readily replaced by cell-derived ECM in a few days [124]. However, numerous obstacles hinder the wide use of fibrin gel in tissue engineering, including its weak mechanical properties, and the possibility of disease transmission and gel shrinkage [125]. The gel shrinkage can be decreased using fixing agents like poly L-lysine [96]. Mechanical properties of fibrin scaffolds can be easily tailored through adjusting the concentration and ionic strength of fibrinogen to obtain gel viscoelastic properties mimicking those of native ECM, promote the diffusion of nutrients and metabolites, as well as promote cell encapsulation [123]. In addition, the modification of the fibrin polymerization process could adjust the polymerized gel’s mechanical properties through modifying the fiber thickness, degree of porosity and branching of the formed gel [126].

Fibrin and its degradation by-products lack any anti-bacterial activity, which could represent a drawback for the use of fibrin hydrogel in REP [123], since residual microorganisms in the canal space and dentinal tubules may hinder dentin–pulp complex regeneration [127]. The persistence of residual bacteria in the canal space after the disinfection step could be attributed to bacterial organization into biofilms on the dentin surface, making them innately more resistant to anti-microbial agents and deep penetration into the dentinal tubules. Residual bacteria affect dentin–pulp complex regeneration through triggering the host immune/inflammatory response [82]. The incorporation of anti-bacterial agents into the fibrin scaffold could represent a viable solution for such a limitation [123]. Other natural hydrogels, including chitosan-based ones, are known to possess anti-microbial activity against a variety of Gram-negative and Gram-positive bacteria, including Enterococcus faecalis (*E. faecalis*) [128,129], which are highly resistant to endodontic disinfectants and are frequently found in persistent intra-radicular or extra-radicular infections [130].

Thus, an injectable fibrin–chitosan hydrogel, composed of 10 mg/mL fibrinogen and 0.5% (*w*/*w*), 40% degree of acetylation chitosan, with physiological pH (≈7.2), was introduced. Enriching the fibrin-hydrogel with chitosan proved to be efficient in imparting anti-bacterial properties to the fibrin hydrogel, evident by the reduction in *E. faecalis* bacterial growth in contact with the hydrogel, as compared to the unmodified fibrin hydrogel [123]. Fibrin–chitosan cellularized hydrogels, encapsulating DPSCs, showed similar results to the unmodified fibrin hydrogel in terms of in vitro cellular viability and fibroblast-like morphology. Both hydrogel groups also exhibited similar DPSCs proliferation rates (detected by the expression of nuclear marker MKI67) and type I/III collagen production capacities, both detected by RT-qPCR and immunohistochemistry at the gene and protein level. Thus, the blending of chitosan in fibrin hydrogels would be a promising construct for REP, imparting an anti-bacterial effect without compromising the excellent biocompatibility of fibrin [123].

Still, a fibrin–chitosan hydrogel did not modify the early fibrin-triggered inflammatory response in the amputated dental pulp tissue in an in vivo model of rat incisor pulpotomy, as compared to the unmodified hydrogel. Both groups revealed a strong increase in interleukin-6 (IL-6) transcript in the dental pulp when compared to the dental pulp of untreated teeth. The leukocytes percentage was similar in all groups, as evaluated by fluorescence-activated cell sorting; however, the neutrophil granulocytes proportion in the leukocyte population increased in the dental pulp/hydrogel interface in both hydrogel groups. Using triple sequential immunofluorescence staining, M2 macrophages, but not M1, were clearly detected in the dental pulp close to this zone of neutrophil infiltration. This suggests that both hydrogels could promote the polarization of pro-regenerative M2 macrophages and that fibrin–chitosan hydrogel is a promising candidate for vital-pulp therapies [131].

Antibiotic-loaded nanoparticles (NPs) are also considered efficient for the eradication of bacterial biofilms, as they have the ability to deeply penetrate into biofilm matrices, thus improving the delivery of antibiotics to even the deepest and most persistent bacteria [132]. Fibrin hydrogel NPs incorporating free clindamycin (CLIN) or CLIN-loaded poly (D, L) lactic acid (PLA) demonstrated anti-bacterial effects against *E. faecalis* in a dose-dependent manner. Both groups also inhibited the formation of an *E. faecalis* biofilm. Thus, CLIN loading into PLA-NPs did not affect CLIN anti-microbial properties, and the NPs restrained CLIN release from the hydrogel as compared to when added in a free form [82]. NPs are known to ensure a high antibiotic concentration locally through protection of the drug structure, enhancing its bioavailability and biodistribution [133]. These results suggest that the CLIN-PLA-NPs allows the maintenance of the CLIN within the nanocomposite hydrogels in comparison to free CLIN (only tested for one day) [82]. DPSCs’ viability and Col I synthesis in the cellularized hydrogel were similar to other hydrogel groups containing free CLIN, empty nanoparticles, or fibrin alone with no NPs. This suggests that the incorporation of CLIN-PLA-NPs did not affect the biocompatibility of the fibrin hydrogel [82].

The in vitro co-culturing of DPSCs and HUVECs in fibrin hydrogel loaded with DPSCs-derived extracellular vesicles (EVs) resulted in enhanced rapid neo-vascularization under starvation culture, without any exogenous GFs, to mimic the harsh environment of nutritional deficiency during pulp regeneration [134]. A higher frequency of apoptosis was also reported with co-cultures in EVs-loaded fibrin gel after five days. It was postulated that EVs stimulated the encapsulated cells to secrete VEGF in order to support rapid neo-vascularization [71,134,135]. Fibrin gels were thereby evidently able to retain and preserve the EVs’ activity. DPSCs-derived EVs, labeled with Cell Mask Green dye, were internalized by cultured HUVECs, which ensured the delivery of the encapsulated GFs without exposure to the extracellular environment, thus protecting such vulnerable bioactive molecules [71].

#### 4.1.4. Matrigel

Matrigel is a biologically active, sterile extract of basement membrane proteins derived from Engelbreth–Holm–Swarm mouse sarcoma. This type of tumor is characterized by having abundant ECM [136,137]. It is mainly composed of laminin, Col IV and entactin, in addition to a variable amount of basic FGF, epidermal growth factor, IGF-1, connective tissue growth factor and several transcription factors [138,139]. A growth factor-reduced version of Matrigel is also available [140]. Matrigel is commercially available as a frozen protein to be diluted in PBS, and can self-assemble into a hydrogel at 37 °C [137]. Owing to its natural origin, Matrigel represents an excellent matrix for stem/progenitor cell culture that can mimic the ECM, promote cell interaction [138] and support the differentiation of many cell types [136]. Moreover, Matrigel can maintain SCAP viability [141]. Unfortunately, being driven from the tumor and due to batch-to-batch variabilities that may affect its mechanical and biochemical properties, there are important factors that hinder its use [138].

Investigations demonstrated that Matrigel exerts a positive effect upon culturing with dental stem cells. DPSCs transfected with the VEGF gene cultured on Matrigel promoted endothelial cell migration and vascular-tube formation [142]. Additionally, DPSCs cultured on Matrigel along with FGF-2 and TGF-β1-releasing poly (glycolide-co-lactide) microspheres showed controlled growth factor release, which promoted stem/progenitor cells’ proliferation and migration in vitro [143]. Rat bone marrow mesenchymal stem cells (BMMSCs) suspended in Matrigel hydrogel were loaded on porous poly (L-lactic acid) (PLLA) scaffold before implantation into pulpotomized rats’ maxillary molars. Cellular pulp-like tissues were successfully regenerated with no signs of inflammatory response [77,78,79,80]. CD68+ newly recruited macrophages, with distinct phagosomes, were detectable around the scaffolds, and nestin-positive odontoblast were observed lining the pulpal surface of dentin near the implantation site, with strong DSPP expression confirming odontoblasts differentiation [77]. Dentin bridge formation was also detected [79]. This evident regeneration was attributed to BMMSCs, which survived and colonized within the implantation site and were able to differentiate into odontoblasts [80]. Loading Matrigel hydrogel with rat dermal microvascular endothelial cells, in addition to BMMSCs in the previous model, yielded a thicker dentin bridge, more organized pulpal tissue formation, and the upregulation of DSPP, nestin and pro-angiogenic factors’ expression, including Bcl-2, Cxcl1, Cxcr2 and VEGF expression [78]. Furthermore, a shift in the macrophage population from phagocytic M1 phenotype to pro-reparative M2 phenotype was evident during pulp regeneration [79].

#### 4.1.5. Keratin Hydrogel

Keratin is a natural structural fibrous protein associated with epithelial cells and skin appendages. It includes hard keratin occurring in hair, wool, hooves, claws and finger nails, in addition to soft keratin, which covers the epithelial surface [144,145]. Hard keratin is formed from tightly packed filaments in the cysteine-rich proteins matrix, while soft keratin is composed of loosely packed filaments [144,146]. Keratin is considered as one of the most abundant natural polymers [147]. It is usually extracted under low pH in the presence of reducing agents to give rise to keratein, or of oxidizing, agents giving rise to keratose [145,148].

Extracted keratin proteins can self-assemble into highly porous fibrous scaffolds characterized by a reproducible architecture, which creates a 3D scaffold favorable for cellular attachment and growth [145,149,150]. It is also characterized by having RGD, Leu-Asp-Val (LDV) and Leu-Asp-Ser (LDS) cell-binding motifs which facilitate cellular attachment [151]. Keratin can affect biological cell behavior and properties [152,153], modulate cellular response to apoptotic signals [153], and influence epithelial cells’ polarization. It is implicated in the regulation of innate immunity and epithelial inflammation [154]. Keratin can also regulate epithelial cell migration, proliferation, epithelial barrier formation and function, and wound healing, [152,155]. Furthermore, keratin hydrogel can promote the adhesion, proliferation and differentiation of adipose stem/progenitor cells into adipocytes, osteoblasts, vascular endothelial cells and myocyte in vitro, and enhance cutaneous wound healing in vivo [156].

Keratin-based hydrogels are biocompatible, highly porous, highly hydrophilic and characterized by a high swelling ratio, which can increase the hydrogel pore size and facilitate cellular and nutrient infiltration. Keratin hydrogels also exhibit slow biodegradation in vitro and in vivo, as a result of protein hydrolysis, which promotes tissue regeneration [69,150]. It also has adequate mechanical properties [157]. Additionally, keratin derived from human hair did not elicit an immune reaction [158].

Keratin hydrogels can be beneficial in dentin–pulp complex regeneration as they are associated with the upregulation of the proliferation and differentiation of odontoblasts and odontoblasts-like cells [69,70], as well as DPSCs in vitro [70], which was evidenced by the increased expressions of ALP and late odontoblast marker DMP-1 [69]. An injectable keratin hydrogel applied to rats’ second molar with partial pulpotomy was compatible with pulp tissues. It was associated with the formation of dentin-like material, resembling reparative dentin, along the root canal walls with increased DMP-1 expression. However, the coronal portion of the pulp did not show signs of regeneration, and it was filled with hydrogel remnants along with pulp tissues and poorly calcified material [150]. Therefore, keratin hydrogel constitutes a natural, abundant, biocompatible and biodegradable natural hydrogel that can be used as an injectable stem/progenitor cells scaffold for dentin–pulp complex regeneration.

#### 4.1.6. Alginate

Alginates are naturally derived anionic biopolymers extracted from brown seaweeds. They are unbranched polysaccharides consisting of covalently linked 1,4-linked β-D mannuronic acid (M) and α-L-guluronic acid (G) units, bonded together in different sequences and blocks along the polymer chain, depending on the origin of the alginate. Alginates can be prepared with a wide range of molecular weights (typically 50–100,000 kDa) [159]. Unmodified alginates are non-biodegradable in mammals, as mammals lack the enzyme (i.e., alginase) that can cleave the polymer chains. Alternatively, ionically crosslinked alginate hydrogels disintegrate progressively in vivo due to the release of the divalent cations that crosslink the hydrogel into the surrounding media in exchange with the monovalent cations, such as sodium ions [160]. In addition, the partial oxidation of alginate chains using sodium periodate makes it biodegradable through cleavage of the carbon–carbon bond of the cis-diol group in the uronate residue, and altering the chain conformation, promoting the hydrolysis of alginate in aqueous solutions [161]. Alginates also suffer from low mechanical stiffness and an uncontrolled degradation rate in vivo. Their mechanical strength can be improved through crosslinking using a calcium chloride bath, whereby calcium ions diffuse into the solution and crosslink the alginate [162]; increasing the calcium content and crosslinking density allows tailoring the mechanical properties [96]. Alginate hydrogels are transparent, allowing easy microscopic observation of entrapped cells and easy cell recovery without cellular damage. The use of highly purified and properly characterized alginates avoids the risk of batch-to-batch variation and immunogenic response [94,159]. A major limitation of alginate is its slow biodegradability and its being non-bioactive. These limitations could be overcome by a number of chemical and biochemical modifications [94].

Alginates are commonly used biomaterials for tissue repair, and when used as hydrogels, they can provide an ECM with hydration properties that promote cellular wound healing [86]. They are biocompatible, non-cytotoxic and non-immunogenic. They show good hydrogel-forming properties, whereby the gelation process is carried out using non-toxic solvents and under physiological conditions (in terms of pH and temperature), which provide easy cell encapsulation and entrapment, offering great potential for cell delivery. Alginate hydrogels are soft in nature, mimicking most native tissues [94]. Moreover, alginate has tunable mechanical properties that can be tailored to cover a range of stiffnesses to match various tissues. The tailoring of alginate mechanical properties can be done by varying different parameters, such as the polymer molecular weight, source, concentration and chemical modifications, and/or the type and degree of crosslinking [159].

Alginate hydrogels present a viable matrix for dental regeneration and can be used for the delivery of exogenous GFs or agents used to release endogenous GFs from tissues, thus enhancing the natural regenerative capacity of the dental pulp. The application of TGF-β1 or HCL acid-treated alginate hydrogels to in vitro cultured human tooth slices resulted in reactionary dentinogenic responses, as evident by the increased predentin width [86]. TGF-β1 has long been known as a modulator for the induction of dental pulp cells’ proliferation and odontoblast-like cell differentiation, and the upregulation of ECM secretion by odontoblasts in the human dentin–pulp complex [163,164]. The increased matrix secretion observed with the acid-treated alginate hydrogel could be explained on the basis of a similar mechanism through the release of endogenous TGF-β by the acid solubilization of the dentin matrix. The reparative dentinogenic response evident by the de novo matrix secretion on the cut pulpal surface suggests that TGF-β1, from either the hydrogel containing it or endogenously from dentin, is a potent modulator of odontoblast-like cell differentiation from pulpal progenitor cells, besides inducing dentin matrix secretion [86].

The coupling of an entire protein to alginate in order to promote cellular adhesion is difficult to control, as it may trigger an unwanted immune response that causes proteolytic degradation. Hence the immobilization of cell recognition motifs, such as RGD peptide, is very common [165]. The RGD tripeptide motif represents the minimal essential cell adhesion peptide sequence associated with integrins in cell surface membranes. RGD is found in various ECM proteins, such as collagen, fibronectin, vitronectin, laminin and osteopontin [94,165]. Alginate hydrogels modified by RGD the peptide sequence promote cell adhesion, migration, proliferation and differentiation [96]. In addition, the interplay between stem/progenitor cells and vascular endothelium is a key process in early tissue morphogenesis. The generation of a functional vascular network that can reconnect with the native blood supply is crucial for securing proper blood supply for the modulation of neo-tissue formation by the stem/progenitor cells, to supply nutrients and oxygen as well as remove waste products [83]. By the in vitro co-culturing of HUVECs with DPSCs, it has been shown that HUVECs modulate the odonto/osteogenic differentiation of DPSCs. Moreover, DPSCs enhanced the angiogenic potential of HUVECs, as evident from the stabilization of the formed pre-existing vessel-like structures, and increased their longevity [166]. RGD-modified alginate hydrogels created a favorable environment for encapsulating DPSCs and HUVECs. These constructs could also retain and locally release the functional quantities of VEGF and FGF active molecules. The data showed that the combined addition of FGF and VEGF led to increased proliferation of both DPSC and HUVEC in the first 24 h, compared to constructs containing a single morphogen or none at all. The RGD-modified alginate constructs showed a higher retention capacity for FGF-2, as compared to VEGF, with sustained release till day thirteen. On the other hand, the release of VEGF was almost complete within the first seven days. The authors suggested a modification of the constructs to include an exterior polysaccharide coating encapsulating DPSCs, and its optimization to promote the cells’ differentiation into odontoblast-like cells. This coating could also slow down the release of the GFs, thus providing longer sustained release periods [83].

Three percent alginate hydrogel doped with pECM was employed for encapsulating primary DPSCs followed by induced differentiation in mineralizing medium, supplemented with dexamethasone, β-glycerophosphate and ascorbic acid. The data showed a time-dependent mineral deposition at the periphery of the hydrogel, as demonstrated histologically using hematoxylin and eosin, and by micro-CT analysis. Only cells adjacent to the outer surface of the alginate hydrogel with pECM contributed to the formation of mineralized tissue, which could be explained based on the reduced diffusion of mineralization signals into the hydrogel, differences in oxygen tension and/or physical restriction of the more deeply encapsulated cells [81].

Verma et al. [127] investigated the regenerative potential of oxidized alginate–fibrin hydrogel microbeads encapsulating DPSCs compared with traditional revascularization protocol. The results showed no significant differences between both experimental groups in terms of radiographic root development. The presence of residual bacteria was further defined to be detrimental for root development in both groups, as it was strongly associated with decreased root length, decreased apical and mid-root wall thickness and decreased dentin-associated mineralized tissue formation [127]. Conclusively, to promote pulp regeneration, the efficient disinfection of root canal space and dentinal walls is crucial before performing these procedures, with the required degree possibly higher than that required in traditional endodontic therapy [167].

Alginate hydrogel has also been used as a bioink for 3D bioprinting, and for the deposition and patterning of cell-laden 3D constructs [168,169]. Blending alginate hydrogels with dentin matrix components was utilized to construct bioink encapsulating SCAP. Soluble dentin molecules in the hydrogel bioink significantly enhanced the odontogenic differentiation of SCAP, as was evident by the increased ALP protein expression and upregulation of both ALP and runt-related transcription factor (RUNX2), which are osteo/odontogenic lineage-specifying markers [168].

As discussed earlier, alginate per se does not promote cellular adhesion as it lacks cell adhesion motifs [94,160]. To promote cell attachment to alginate hydrogels, blending them with gelatin was widely investigated for tissue engineering applications [160,169]. Hydrogel blends combining alginate with gelatin were also used as a bioink for constructing 3D printed scaffolds. The 3D bioprinting of glutaraldehyde crosslinked alginate and gelatin hydrogels produced a scaffold with enhanced physical and biological properties. This 3D bioprinting allowed for controlling the scaffold porosity interconnection and pore diameter to create a scaffold which mimics the cellular natural environment. The 3D printed alginate/gelatin hydrogel scaffold aqueous extracts enhanced human DPSCs cell proliferation, mineralization and osteogenic/odontoblastic differentiation in vitro, as evident by the increased expression of ALP, OCN and DSPP, as well as enhancing the deposition of bone-like mineralized nodules [169].

#### 4.1.7. Chitosan Hydrogel

Chitosan consists of N-acetyl glucosamine and glucosamine co-polymer units obtained from chitin [170]. Chitin is extracted from crustaceous shells, mollusks, insects, silkworm chrysalides and microorganisms [171]. Chitosan is provided in the form of gel, scaffold and fibers, and their properties depend on their molecular weight and the degree of acetylation. The extraction source and acetylation procedure regulate the polymer’s final properties. Thereby, Chitosan is likely to be used as a biomaterial for dental application due to its anti-microbial, biocompatible properties, bioactivity, and ability to blend with other materials [172]. Notably, multiple studies presented that chitosan-based scaffolds displayed no cytotoxicity toward various cell types, and had good in vitro biocompatibility [173,174,175,176]. The nature of the crosslinking agent can impact chitosan cytocompatibility. Glutaraldehyde crosslinked chitosan was associated with higher cytotoxicity as compared to either non-crosslinked chitosan or genipin crosslinked chitosan [176].

Chitosan hydrogel networks are classified, based on the chitosan crosslinking methodology and preparation, into two categories. Chemically crosslinked hydrogels are irreversible formed by covalent bonding of the chitosan macromers, which can occur through crosslinkers or a photo-polymerization reaction, while physically crosslinked hydrogels develop through physical interactions, such as ionic interactions or secondary interactions [177]. The chemical modifications of chitosan do not change the fundamental skeleton of chitosan, but improve its properties. The possible chemical modifications examined were oligomerization, alkylation, acylation, quternization, hydroxyalkylation, carboxyalkylation, thiolation, sulfation, phosphorylation, enzymatic modifications and graft copolymerization, giving a wide range of derivatives with modified properties for specific use in pharmaceutical, biomedical and biotechnological fields [178].

Biodegradable glycol chitin-based thermo-responsive hydrogel scaffold (GC-TRS) is an injectable formulation, which is easily manipulated due to its unique thermo-responsive sol–gel transition property and inherent biocompatibility. GC-TRS allowed the proliferation and odontogenic differentiation of human DPSCs, as was evident by the expression of odontogenic/osteogenic markers, including DSPP, DMP1, ON and OP. Thus, it is considered as a promising material for pulp and dentin regeneration [179]. Thermo-sensitive hydrogels containing chitosan/β-glycerophosphate displayed great biocompatibility, where cultured DPSCs showed adhesion and vitality. Furthermore, the chitosan/β-glycerophosphate hydrogel maintained a sustained delivery of VEGF, which promoted the odontogenic differentiation of DPSCs more effectively than VEGF only, subsequently highlighting its potential application as a pulp capping material [180]. Another chitosan scaffold containing silver and bioactive glass promoted the in vitro odontogenic differentiation of DPSCs without affecting their proliferation, and downregulated the levels of inflammatory cytokines IL-1β, IL-6, tumor necrosis factor-alpha and IL-8, with the concomitant inhibition of *streptococcus mutans* and *lactobacillus casei* growth [181].

An in vivo study investigated dentin–pulp tissue regeneration, using DPSCs loaded in a chitosan hydrogel scaffold combined with various GFs delivered into non-vital immature permanent teeth with induced apical periodontitis in dogs. Radiographic and histopathological evidence indicated significantly greater radicular thickening, root lengthening, apical closure of the root and the presence of a pulp-like tissue [182].

#### 4.1.8. Hyaluronic Acid Hydrogel

Hyaluronic acid (HA) is a linear polysaccharide composed of alternating units of repeating disaccharide, D-glucuronic acid and N-acetyl-D-glucosamine, linked together via alternating β-1,4 and β-1,3 glycosidic bonds. It is a non-sulphated glycosaminoglycan found in the ECM of many soft connective tissues [183]. HA is biocompatible, biodegradable, bioactive, non-immunogenic and non-thrombogenic, and has high water affinity [184]. HA plays an important role in maintaining morphologic organization by preserving extracellular spaces and anti-oxidant effect, is a strong inflammation mediator [185] and activates various signaling pathways [186]. In light of the above, HA represents an outstanding candidate for tissue engineering [187,188] and drug delivery systems [189].

In physiological environments, HA is subjected to multiple degradation processes, including hydrolysis and enzymatic digestion, via the hyaluronidase enzyme. Therefore, in order to control the degradation rate and improve its mechanical properties, crosslinking or conjugation strategies have been used to stabilize HA and at the same time to preserve their native biological functions [190].

Interestingly, the dental pulp contains large amounts of glycosaminoglycans [191]. HA was found to contribute to the initial development of the dentin matrix and dental pulp [192]. Injectable HA hydrogel, crosslinked with 1, 4-butanediol diglycidyl ether, has shown great potential as a scaffold for dentin–pulp complex regeneration. In vivo, this hydrogel was combined with BMP-4 and loaded with dental mesenchymal cells, then injected subcutaneously in nude mice. A typical dentin-like structure was observed having columnar odontoblast-like cells with polarized basal nuclei and blood vessels. These cells were aligned against the regenerated dentin-like tissue, while the dentinal tubules were arranged radially from the pulp-like tissue [193]. Another injectable HA hydrogel, based on hydrazone crosslinking between hydrazide and aldehyde groups, reinforced with cellulose nanocrystals and enriched with human PL, was examined for endodontic regenerative therapies. Since HA plays a crucial role in stimulating wound healing and tissue regeneration through increasing cellular proliferation and regulating angiogenesis, this hydrogel was suggested to be suitable as a supportive matrix for cell culture and promoting cell sprouting. This hydrogel also acted as a GF-controlled delivery system through its sustained release of chemotactic and pro-angiogenic GFs. Together with the fact that this hydrogel can be injected easily into any desired defect and crosslinked in situ, this clearly endorsed it for regenerative dentistry [194].

A photo crosslinkable hyaluronic acid/platelet lysate (HA/PL) hydrogel system was developed by modifying HA with methacrylic anhydride and curing by ultraviolet light exposure to create a further hydrogel for dentin–pulp repair. PL delivers multiple cytokines and GFs involved in wound healing, which could help HA hydrogels to increase pulp cell proliferation. HA/PL hydrogels successfully recruited cells from the cell monolayers of human DPSCs obtained from permanent teeth. Following odontogenic induction, human DPSCs seeded on HA/PL hydrogel showed the most abundant calcium deposits, with increased metabolic activity and proliferation of human DPSCs at all time points. These results provided clear evidence of the proposed system’s potential as a promising repair system in dentin–pulp damages [195]. Furthermore, chemically conjugated methacrylate HA hydrogel was verified to be non-toxic to human DPSCs, as proven by methyl thiazolyl tetrazolium (MTT) assay, and was able to retain the stemness of human DPSCs with increased expression of NANOG and SOX2 though maintaining native stem cell shape [196]. Similarly, in a mini swine model, pulpectomized root canals were regenerated with vascularized pulp-like tissue, and accumulated with newly generated dentin-like tissue or osteodentin against the canal walls following transplantation with autologous and allogeneic swine DPSCs carried in a HA hydrogel [197].

Restylane, a Food and Drug Administration (FDA)-approved HA-based injectable hydrogel, is considered one of the most reported biopolymers for endodontic applications. Restylane was examined for its use as a scaffold for REP in vitro. This HA hydrogel promoted SCAP survival, mineralization, and odontoblastic differentiation. In comparison to Matrigel, which revealed significantly declined cell viability after 24 h as a result of Matrigel degradation, Restylane promoted greater ALP activity and upregulated the expressions of DSPP, DMP-1 and MEPE [198].

#### 4.1.9. Agarose Hydrogel

Agarose is a linear component of the natural polysaccharide agar, together with agaropectin, which is obtained from sea algae. Structurally, Agarose is a polymer of agarobiose that consists of repeating units of D-galactose and 3, 6-anhydro-L-galactopyranose. Owing to its neutral, hydrophilic, porous, non-toxic, low-cost and biocompatible properties, it is used widely in biomedicine [199,200]. Agarose can be prepared as a thermal-reversible gel. Agarose’s gelation and melting points change from 30–40 °C to 80–90 °C, respectively, depending on the molecular weight, the concentration and number of its side groups [201,202]. Agarose dissolves in water, forming a gel with a rigid net forming a 3D plastic and porous reticulum. Agarose gel appears as an apyrogenic, colorless, transparent gel, and is viscous-elastic at temperatures above 45 °C. Thus, specific treatment is needed for it to be extruded from the needle at 25 °C (room temperature). Agarose is resorbed slowly by means of phagocytosis, as macrophages produce α-galactosidase enzymes that cause polymer destruction [203,204,205,206].

The Agarose gelation process occurs in three consecutive steps: induction, gelation and pseudo-equilibrium. Hydrogen bonding and electrostatic interaction give rise to the helical structure of the Agarose molecule-forming gel [207,208]. Thereby, Agarose hydrogels are made without using toxic crosslinking agents [209,210], making it a biocompatible polymer [211]. Additionally, Agarose is used massively in biomedical applications owing to its controlled self-gelling properties, water-solubility, adjustable mechanical properties and non-immunogenic properties. Agarose has an adjustable water adsorption capacity, which provides the cells with a suitable microenvironment for cellular activity [208]. It has been used also to investigate mechanical load reactions for chondrocytes and mesenchymal stem cells [212], and in enamel regeneration [213].

Agarose hydrogel provided a microenvironment for dentin remineralization and enamel regeneration. Human-derived dentin slices were covered with ionic CaCl_2_ and non-ionic agarose hydrogel to occlude the exposed dentinal tubules and regenerate an enamel prism-like tissue on the dentin surface. Scanning electron microscopic (SEM) images demonstrated that crystals occluded the dentinal tubules. The densely packed growth mode along the long-axis enabled the crystals to align themselves in parallel to each other, to form an approximately 2.5 mm enamel prism-like layer on the dentin surface. Moreover, radiographic diffraction tests confirmed the hydroxyapatite nature of crystals. This could be an alternative therapeutic technique for the management of dentin hypersensitivity [214].

#### 4.1.10. Cellulose Hydrogel

Cellulose is a fibrous, water-insoluble element found in plants, some animals (e.g., tunicates), fungi, and few bacteria. Cellulose hydrogels can be acquired through either the physical or chemical stabilization of cellulose solutions. Hydrogels can be obtained either directly from native cellulose or from cellulose derivatives, including methyl cellulose (MC), hydroxypropyl cellulose (HPC), hydroxypropylmethyl cellulose (HPMC) and carboxymethyl cellulose (CMC). In physical crosslinked gels, there is no covalent bonding formation or breakage, and the crosslinked network is formed through ionic bonding, hydrogen bonding, or an associative polymer–polymer interaction [215]. Physically crosslinked hydrogels are used as scaffolds for cell cultures, in cartilage models and as implants in bone defects [216], while chemical crosslinked hydrogels are prepared through crosslinking two or more kinds of polymer chains, either under ultraviolet irradiation [217] or with a functionalized crosslinker [218]. Cellulose-based composite hydrogels are made by mixing natural biodegradable polymers or synthetic polymers with cellulose to obtain composite hydrogels [219,220].

Since physically crosslinked hydrogels are reversible [221], they might flow under given conditions (e.g., mechanical loading), and might degrade in an uncontrollable manner. Owing to these shortcomings, physical hydrogels based on MC and HPMC are not recommended for use in vivo. On the contrary, MC hydrogels have been suggested as cell sheet harvest systems in vitro [222].

Chemical modifications for the cellulose backbone might be done before crosslinking to obtain stable hydrogels with given properties. Silated-HPMC has been developed, which crosslinks through condensation reactions upon decreasing the pH in water solutions [223,224]. Such hydrogels have the potential for both in vivo injection and in vitro culturing. They can be used as a scaffold in 3D cultures of osteogenic cells [225]. A Silated-HPMC hydrogel scaffold was able to extract and release non-collagenous matrix proteins from dentin powder, particularly TGF-β1. Thus, a hydrogel containing endogenous growth factors and morphogens native to dentin is likely an important adjuvant in clinical REP [226].

The addition of hydroxyapatite nanoparticles to a CMC hydrogel stimulated the differentiation of DPSCs to the osteoblastic and odontoblastic lineage. This was supported by the upregulated expression of osteogenic markers ALP, RUNX2, COL-IA1 and OP, and odontogenic markers DMP-1 and DSPP, after 21 days of culture. Moreover, the DPSCs seeded on CMC-HA showed good biocompatibility, adhesion and viability. This indicates that DPSCs and CMC-HA hydrogel could be considered as promising candidates for dentin–pulp complex and periodontal tissue regeneration [227].

In an in vitro system, a recent thermo-responsive injectable formulation, which is composed of BAG (45S5 Bioglass^®^) nanoparticles, Pluronic F127 and HPMC, has been investigated for potential dental application. This formulation was flowable, injectable at room temperature, dispersible at 4 °C and hardened at body temperature in 10 to 30 min. The injectable bioactive glass was completely biocompatible, as assessed by MTT assay, with favored surface morphology, chemical structure and osteoinductive/osteoconductive properties that promoted osteoblast proliferation and growth significantly. The hydrophilicity and low surface energy of the HPMC and Pluronic F127 made the cells more liable to attach to the scaffold, and showed enhanced cell proliferation. Moreover, together they showed the ability to regenerate dentin in prepared cavities in extracted bovine teeth [228].

#### 4.1.11. Extracellular Matrix Hydrogels from Decellularized Tissues

Decellularized scaffolds have become prevalent in the tissue engineering field. The first reported production of decellularized ECM from a tissue source to be used as a bioscaffold was the small intestinal submucosa in vascular applications [229,230,231,232,233]. The primary studies removed cells, while preserving the structural and functional proteins of the ECM, such as glycosaminoglycans, proteoglycans and GFs [234]. Thereby, the obtained materials retain the biochemical complexity, nanostructure and bioinductive properties of the native matrix [235]. ECM-derived materials are FDA-approved and have been used in millions of patients [235,236].

ECM bioscaffolds can be transformed into injectable hydrogels in two main steps. ECM hydrogel can be formed by enzymatic digestion mainly by pepsin-mediated solubilization. Then, ECM is transported to physiological pH and salt conditions to match in vivo conditions, as well as to inactivate pepsin. The digested ECM self-assembles into a nano-fibrous hydrogel upon incubation at 37 °C or injection in vivo [237,238]. The other approach is ECM homogenization using mortar and pestle, or high-speed shear mixing within a high salt buffer that physically disrupts the ECM particles and collagen fiber structure at physiologic pH [238,239,240,241,242]. SEM images of fully-formed ECM hydrogels reveal a loosely arranged nano-fibrous scaffold with interconnecting pores [243].

ECM hydrogel drawbacks include increased manufacturing time, and adding foreign protein for digestion. However, ECM hydrogels are widely used for various tissue engineering applications, owing to their ability to form an injectable hydrogel and their in vivo safety for any clinical application, thus providing a new minimally-invasive procedure for regenerative medicine [244].

A porcine urinary bladder matrix hydrogel containing bioactive glass with silver ions (Ag-BG/ECM) was investigated for dental pulp regeneration [245,246]. Ag-BG/ECM demonstrated enhanced anti-bacterial and regenerative properties. This fabricated hydrogel provided a better chemical composition and microenvironment that promoted DPSCs proliferation and differentiation potential, as well as the inhibition of apoptosis in vitro [245,246], while via in vivo implantation into the dorsum of mice, Ag-BG/ECM induced pulp–dentin complex formation. The formed tissues presented a dentin-like morphology, with the collagen matrix deposited perpendicular to the odontoblast-like layer. These cells have the phenotypic characteristics of odontoblasts expressing nestin and DSP [245].

The human decellularized dental pulp matrix (DDPM-G) is fabricated by consecutive decellularization and sol–gel transition at 37 °C. In vitro, the human DDPM-G promoted human DPSCs attachment, cell migration and proliferation. Besides this, the human DDPM-G-coated surfaces aided the induced human DPSCs’ differentiation into odontoblast-like and neural-like cells. Overall, the human DDPM-G holds tremendous translational potential in dental pulp regeneration, due to the ease of application, whereby the decellularized dental pulp contents are injected into the root canals and undergo gelation in situ. Therefore, it could serve as a carrier for exogenous stem cells due to its high compatibility [247]. Additionally, the injectable human-treated dentin matrix and alginate have demonstrated potential as pulp-capping agents clinically [248]. Studies discussing natural hydrogels used for dentin-pulp complex regeneration are summarized in Table 1.

### 4.2. Synthetic Hydrogels

Synthetic hydrogels are a group of materials with various biomedical applications. They are characterized by a high water absorption ability, improved tunable mechanical properties, thermostability and durability as compared to natural hydrogels, and are relatively inexpensive [31,251,252]. They feature different properties and characteristics that vary and can be tailored according to their chemical structure, crosslinking and method of fabrication [252,253]. Unfortunately, many synthetic scaffolds give rise to acidic byproducts upon degradation, which can elicit inflammation upon in vivo implantation [254]. Synthetic hydrogels can be divided into degradable hydrogels, such as PLA-based hydrogels, and non-degradable hydrogels, such as PEG.

#### 4.2.1. Poly Lactic Acid-Based Hydrogels

Poly Lactic Acid (PLA) is a biocompatible, biodegradable, hydrophobic, biobased synthetic polymer obtained through the bacterial fermentation of renewable, plant-based carbohydrates. It has adequate mechanical properties, and its degradation byproducts are carbon dioxide and water, which are safe and nontoxic. PLLA is a commonly used isomeric form of PLA [255,256]. PLA-based scaffolds can upregulate DPSCs differentiation in addition to ALP, OCN, BSP, COL-I and DSPP expression in vitro, and enhance dentin formation in vivo [257]. PLA nanoparticles can be used as antibiotic carriers for reducing bacterial load for dental tissue regeneration [82]. PLA nanoparticles incorporating clindamycin loaded on fibrin hydrogel demonstrated a potent anti-bacterial effect without negatively affecting the DPSCs’ vitality or function [82].

PLA can be conjugated with hydrophilic synthetic or natural polymers, such as PEG and polysaccharides, respectively, giving rise to hydrogels [252,255]. The PLA-PEG co-polymer is characterized by being soluble at room temperature and gel at body temperature, it is also biodegradable, biocompatible, constitutes an excellent carrier for drug delivery, and can enhance DPSCs adhesion and proliferation as well as the expression of odontoblasts-related markers ALP, DMP1, DSPP, COL-I and OPN in vitro [252,258,259]. Polylactic‑polyglycolic acid (PLGA) and PEG co-polymer-based hydrogel were successfully used clinically for tooth apexogenesis and dentin regeneration [260].

#### 4.2.2. Polydimethylsiloxane Hydrogels

Polydimethylsiloxane (PDMS) is one of the polymeric organosilicon compounds that are frequently ascribed to silicones [261]. Owing to its inert, nontoxic effect and stiffness versatility, PDMS is the most vastly used silicon-based organic polymer [262].

Taking into consideration that physical properties can affect the differentiation of stem/progenitor cells, the influence of the stiffness of PDMS on the behavior of DPSCs was investigated. Different ratios of liquid oligomeric base and curing agent (10:1, 20:1, 30:1 and 40:1) were utilized to form PDMS substrates with different stiffness properties (135 kPa, 54 kPa, 16 kPa and 6 kPa, respectively) [263]. PDMS was treated with dopamine solution in order to increase its cell adhesion potential. SEM results revealed that soft substrates changed the cell morphology and inhibited DPSCs’ proliferation. Following conversion into osteogenic or odontogenic media, the expression level of osteogenic and odontogenic markers was positively correlated to the substrate stiffness, comprising ALP, OCN, OPN, RUNX-2, BMP-2, DSPP and DMP-1. The results of a Western blot analysis of the two important members of the Wnt signaling pathway, the glycogen synthase kinase-3β (GSK-3β) and β-catenin proteins, revealed that the mechanical properties promoted the function of DPSCs related to the Wnt/β-catenin pathway, as β-catenin was significantly upregulated in DPSCs growing on stiffer substrates, which would affect the osteoblast, chondrocyte and odontogenic differentiation potential of DPSCs [264,265,266].

#### 4.2.3. Poly-N-Isopropylacrylamide Gel

Pulp regeneration using the tissue engineering approach is challenging, since scaffolds increase the risk of inflammation and infection. To overcome that, an attempt to transplant scaffold-free 3D cell constructs capable of forming pulp-like tissue derived from DPSCs was carried out. Rod-shaped 3D DPSCs constructs were fabricated by shaping sheet-like aggregates of DPSCs with a poly-N-isopropylacrylamide gel mold prepared through computed tomography image design [267]. Poly-N-isopropylacrylamide is a hydrogel with thermo-reversible gelation properties. It gels at temperatures ranging from 32 to 35 °C, and reverts into a sol upon cooling [268,269]. To induce odontoblastic differentiation, DPSCs hydrogel constructs were cultured with odontoblastic differentiation media. In vitro results showed that DPSCs within the constructs remained viable even after prolonged culturing for 20 days. The regeneration ability of the construct was evaluated in a circumfluent space to mimic the therapeutic environment. An entire human tooth root was transplanted into the subcutaneous area of immunodeficient mice after being filled with the DPSCs constructs and sealed with the glass ionomer cement. Six weeks after implantation, pulp-like tissues rich in blood vessels were identified in the root canal, where the transplanted DPSCs differentiated into odontoblast-like cells at areas in proximity to dentin. This is attributed to the fact that dentin is a source of GFs (e.g., BMP-2 and TGF-β) [9,270]. Furthermore, immunofluorescent human CD31-positive endothelial cells were evident at the lumen of the regenerated tissue, which proves the anastomosis of the newly formed blood vessels with those of the host without requiring scaffolds or GF. The 3D constructs exhibited a self-organizing ability in vivo and in vitro. The self-organizing ability was evident in vitro through an expression of DSPP mRNA localized in the outer layer of cell constructs, while the expression of NANOG, a stem/progenitor cell marker, was significantly increased in DPSCs in the center of the constructs. The mineralization potential evaluated by ALP activity at the tenth day of culture indicated that DPSCs in the outer layer of the constructs differentiated into odontoblast-like cells, whereas DPSCs in the inner layer maintained their stemness properties [267].

#### 4.2.4. Polyethylene Glycol

PEG is a synthetic polymer that is widely used to produce constructs for biological applications, owing to its unique properties, such as hydrophilicity, non-toxicity, low protein adhesion and non-immunogenicity [271,272,273]. In order to form a hydrogel, PEG must be crosslinked to attain a high water content construct. Additionally, the end hydroxyl groups of PEG molecules can be readily functionalized by groups, such as thiol, carboxyl and acrylate, or attached to other molecules or bioactive agents [43]. PEG-based hydrogels can be synthesized by the radiation crosslinking of PEG or via the covalent crosslinking of PEG macromers with the reactive chain ends [262].

PEG–maleate–citrate (PEGMC) was synthesized using PEG, (MW 200–1000 Da), maleic acid (MA) and citric acid. PEGMC hydrogel proved to be biodegradable, elastomeric, biocompatible and have light-curing crosslinking properties [274]. The efficacy of PEGMC hydrogel as an injectable light-cured drug delivery system for direct pulp capping has been investigated [275]. A pre-hydrogel solution was manufactured using PEGMC (45% *w*/*v*), acrylic acid (AA) crosslinker (5% *w*/*v*), 2,2′-Azobis (2-methylpropionamidine) dihydrochloride (AAPH) photo-initiator (0.1% *w*/*v*) and deionized water. Using a visible light-curing system, various volumes of the pre-hydrogel solution (50, 100, 150, and 200 μL) were used to measure the gelation time required for the photopolymerization of the solution to attain poly-PEGMC (PPEGMC) gel. A dose-dependent cytotoxicity experiment was used to evaluate the effects of varying concentrations of the polymerized hydrogel and its components on cell viability. The results revealed that the gelation time and the light-curing time for the hydrogel were comparable to composite resin. Light exposure for 30 and 60 s resulted in cell viability of 92.5% and 79.7%, respectively, while upon exposure to 90 s of light, the cell viability significantly decreased to 48.9%. For the hydrogel system, cell viability remained up to 80% after a period of 6 h. Moreover, controlled Ca^2+^ release was attained from the calcium hydroxide-enclosed hydrogel, as quantified using a calcium reagent and spectrometer at 550 nm. Simulating the clinical conditions, the injection ability and viscosity into plastic root canal blocks were confirmed for both mandibular and maxillary teeth in a dental model [275]. From these results, it could be deduced that the light-cured PEGMC hydrogel could represent a promising drug delivery vehicle for regenerative endodontics and vital pulp therapy.

#### 4.2.5. Synthetic Self-Assembling Peptide Hydrogel

As discussed earlier, synthetic polymers provide highly tunable material properties, and are relatively inexpensive, biocompatible and biodegradable, however they fail to mimic the complex physiological functions of the native tissue. Collagen, as one of the most commonly used natural biomaterials, mimics natural ECM structurally, but it is expensive, difficult to process and suffers from immunogenicity and purity issues. Thus, novel synthetic or semisynthetic materials, including self-assembling peptide nanofibers, are under development in an attempt to overcome the deficiencies in these materials. They are considered promising candidates for future regenerative therapies, particularly REP, owing to their microstructure and the easier control of their synthesis routes. In addition, they form hydrogels with tunable viscoelastic properties, which are injectable, biocompatible, biodegradable, and can be modified by incorporation of bioactive molecules and motifs for enhanced biological performance [1]. Owing to their synthetic nature and controlled physicochemical properties, self-assembling peptides were described as a promising environment for pulp regeneration [276].

##### Peptide Amphiphiles

Peptide amphiphiles are synthetic self-assembling peptides designed and structured to incorporate both hydrophilic and hydrophobic residues. They can self-assemble in solution into nanofibers under specific conditions with numerous potential applications in regenerative medicine and drug delivery [277]. Peptide-based hydrogels are biocompatible, biodegradable, tunable and can be biofunctionalized to mimic the ECM [278,279]. Additionally, they do not require crosslinking, and therefore, cells and proteins can be added to the peptide without fearing their destruction due to exposure to chemical crosslinking. Unfortunately, they have low mechanical strength [7].

SHED and DPSCs were cultured in the self-assembling nanofiber peptide amphiphile, modified by incorporation of RGD and an enzyme-cleavable site. The hydrogel scaffold was biodegradable, biocompatible, easily handled, could be injected into small defects and it supported stem/progenitor cell proliferation, differentiation and mineralization in vitro [74].

The peptide hydrogel concentration can impact the hydrogel’s physical as well as mechanical properties, and can influence cell behavior as well. Low concentrations of amphiphilic peptide hydrogels are more fluid than high-concentration hydrogels, which facilitate cellular connection. However, they are not steady, have poor mechanical properties and cannot establish a 3D environment for cellular growth. On the contrary, higher concentrations have low fluidity, which renders them difficult to be injected. The proper concentration was estimated at 0.25% hydrogel [280].

##### RADA16-I Peptide Hydrogels

RADA 16-I is a self-assembling synthetic peptide amphiphile composed of 16 amino acid residues. It consists of repeated segments of hydrophobic alanine and hydrophilic arginine, and aspartic acid amino groups [281]. RADA16-I self-assembling synthetic peptide amphiphile hydrogels, loaded with human DPSCs and umbilical cord mesenchymal stem/progenitor cells, were biocompatible, did not affect cell viability and supported cell proliferation particularly at lower (0.25%) hydrogel concentrations. The hydrogel created a 3D environment which supported odontoblastic differentiation following BMP induction, and induced an increased expression of DSPP, DMP-1, ALP and OCN, in addition to increased mineralization. It was concluded that the co-culture of two cell types induced cellular interaction, which enhanced odontogenic differentiation in vitro [280].

Self-assembling RADA16-I peptide hydrogels can be modified by the addition of bioactive molecules to enhance dentin–pulp complex regeneration [87,88]. Self-assembling peptide hydrogel RADA16-I, incorporated with stem cell factor and loaded with DPSCs and HUVECs, was effective in promoting cell adhesion, proliferation, migration and angiogenesis in vitro [87]. Injectable self-assembled peptide hydrogel modified through the incorporation of dentonin sequence, a bioactive component of ECM phospho-glycoprotein, was biocompatible and supported DPSCs proliferation and mineralization in vitro [88].

Puramatrix^TM^ is a commercial RADA16-I self-assembling peptide formulated as an injectable aqueous solution that instantly polymerizes, forming a biodegradable scaffold of nanofibers, when exposed to physiologic concentrations of salts. Puramatrix^TM^ has been tested in inducing the differentiation of primary cells and stem cells [282,283]. Puramatrix^TM^ has yielded promising results for cardiac [284,285,286,287], neural [283,288], hepatic [289], bone [290] and murine calvaria [290] regeneration. The potentiality of Puramatrix^TM^ as an injectable scaffold that is compatible with DPSCs growth and odontoblastic differentiation was investigated. DPSCs were grown in 0.05–0.25% Puramatrix^TM^, and the cell viability and morphology were assessed using the WST-1 assay colorimetrically and by confocal microscopy. Verifying DPSCs’ differentiation into odontoblast-like cells was investigated by measuring the expression of DSPP and DMP-1 upon loading human tooth slices with Puramatrix^TM^ [291]. An elevated expression of these markers by DPSCs is correlated with the deposition of new dentin [292]. DPSCs exhibited the proliferative and morphological features of healthy cells in all concentrations of Puramatrix^TM^. No difference was observed in the DPSCs’ proliferation rate in relation to the concentration of Puramatrix^TM^. Notably, following 21 days of culturing in dentin slices containing Puramatrix^TM^, DPSCs expressed DMP-1 and DSPP [291]. Therefore, it could be concluded that the dentin may have provided odontoblastic differentiation signaling molecules to the stem cells suspended within Puramatrix^TM^ hydrogel.

Securing early angiogenesis through the application of VEGF [292] or co-culturing with vascular endothelial cells [293] could guarantee a successful outcome in dental pulp regeneration [294]. However, VEGF is an essential coordinator for the remodeling of ECM and angiogenesis, but the resulting blood vessels have the tendency for leakage due to structural incompatibilities [295,296]. In relation to this, stromal-derived factor-1 alpha (SDF-1α) is a strong stimulator of the maturation of blood vessels [297,298], and it recruits pericytes and smooth muscle cells to mature and stabilize the newly formed blood vessels [299]. Therefore, human DPSCs, transfected for the overexpression of VEGF-red fluorescent protein (VEGF-RFP), SDF-1α- green fluorescent protein (SDF-1α-GFP), or both genes, using lentivirus were evaluated for their pulp regeneration potential in vivo and in vitro [142]. The transfected cells revealed an upregulated proliferation rate assessed by cell counting kit 8 (CCK-8) and MTT assays at different culturing times. Furthermore, transfected DPSCs promoted the in vitro migration of endothelial cell and vascular-tube formation in Matrigel. When injecting the modified DPSCs enclosed in Puramatrix^TM^ hydrogel into the root canals of 6 mm tooth pieces and implanting them into immunodeficient mice for four weeks, the DPSCs/SDF-1α + DPSCs/VEGF-mixed group resulted in a significant increase in the length of formed pulp-like tissue, as well as vessel area density, as compared to the DPSCs/VEGF group, which indicated a synergistic effect of these two cells when cultured together. Neo-angiogenesis induced by VEGF and SDF-1α overexpressing DPSCs seemed to be less dependent on sprouting (angiogenesis), but occurred via an increase in the cross-sectional area of blood vessels, as most of the vascular lumens within the regenerated pulp-like tissue were perfused, as clearly shown by Haemotoxylin and Eosin staining. Immunohistochemical staining by human CD31 showed only focal single cell positives, rather than circular positives, around the lumens [142]. This was attributed to the formation of the vessel lumen by a combination of host endothelial cells and DPSCs-differentiated endothelial cells [276,300]. Accordingly, an enhanced area of vascularized pulp could be attained in vivo using combined VEGF and SDF-1α overexpression in DPSCs. RGD was modified by incorporating the two functional peptides PRG and KLT (RGD-mimicking peptide and VEGF mimetic peptide sequence, respectively), which further provided a 3D environment favorable for DPSCs and HUVECs adhesion and proliferation, besides angiogenic and odontogenic differentiation in vitro, and stimulated dentin–pulp complex regeneration in vivo following implantation in rats’ pulpotomized molars [89].

Prevascularization of the cell/scaffold construct by endothelial cells is another attempt to develop a novel strategy to trigger angiogenesis in the engineered pulp tissues through rapid anastomosis between the bioengineered cellular/tissue construct and the host vasculature [301,302]. Nevertheless, endothelial cells are sensitive and usually undergo apoptosis in the absence of angiogenesis-related factors, following their incorporation into scaffold materials [303]. Therefore, a 3D culture system that mimics the natural cell milieu, by providing cell–cell interactions-related signaling molecules independent of the used scaffold, may clarify the role of HUVECs interactions during angiogenesis [276]. Commercially available HUVECs, DPSCs extracted from human sound third molars, or co-cultures of different ratios (3:1, 1:1, or 1:3) of both cell types were encapsulated in 3D Puramatrix^TM^ using the Transwell culture model, whereby HUVECs incorporated in Puramatrix^TM^ were layered on a monolayer of DPSCs. Despite the lack of exogenous angiogenesis-related factors, the peptide nanofiber enhanced in vitro cell survival, migration and capillary formation, monitored by confocal microscopy for two weeks. It was postulated that DPSCs promoted early vasculature formation by enhancing the expression of VEGF and the migration of HUVECs. DPSCs/HUVEC co-enclosed Puramatrix^TM^ exhibited vascularized pulp-like tissue with spots of osteodentin after being injected into the root canals of root segments and transplanted into mice for four weeks. The protein level of ALP and von Kossa staining for mineralization revealed that the co-cultured groups (Puramatrix^TM^ + 3:1 DPSCs:HUVECs, and Puramatrix^TM^ + 1:1 DPSCs:HUVECs) exhibited more mineralization than the DPSCs monocultures in vivo. Moreover, immunohistochemical analysis for human-specific CD31, nestin and DSP revealed that the co-cultured groups demonstrated more ECM and vascularization as compared to other groups [276]. Thus, it could be concluded that despite DPSCs being key players in initial angiogenesis, co-ordination between HUVECs and DPSCs is needed to accomplish a balance between angiogenesis, ECM deposition and mineralization on the nanofibrous peptide scaffold, in order to attain the efficient promotion of the cell–cell interactions and cellular cross-talk required for tissue regeneration.

The ability of SHED to construct a functional dental pulp when injected into root canals suspended in Puramatrix^TM^ gel or recombinant human Collagen (rhCollagen) type I was investigated [76]. Upon mixing SHED with Puramatrix™ hydrogel for seven days and with rhCollagen type I for 14 days, and injecting the construct into roots of human premolars, the cells survived and expressed odontoblastic differentiation markers (DMP-I, DSPP, and MEPE) in vitro. Upon implanting the previous constructs subcutaneously into immunodeficient mice, pulp-like tissue with odontoblasts capable of forming neo-dentinal tubules was observed after 35 days from transplantation. The engineered pulp tissue by SHED encapsulated in Puramatrix™ or rhCollagen type I exhibited vascularization and cellularity similar to the control human pulp regarding apoptosis (detected by TUNEL) and neo-dentin deposition, as assessed by confocal microscopy [76]. These data revealed that the latter strategy might facilitate the completion of root formation in necrotic immature permanent teeth.

##### Multidomain Peptides Hydrogels

In 2007, an amphiphilic self-assembling peptide system termed multidomain peptides (MDP) was introduced [304]. The MDP displays distinct domains arranged in an ABA block motif that self-assemble into nanofibers, 6 nm in diameter, with controlled fiber length. The 6 nm diameter of the nanofibers created from MDPs mimics the nanoscale architecture of the natural ECM, whereby cells can bind to the fibers via adhesion molecules, but still interact with other cells in all dimensions [304]. A biodegradable variant of MDP featuring the cell adhesion motif RGD as well as an MMP-2 enzyme-cleavable site for cell-mediated degradation was then introduced [2].

MDP hydrogel has tunable viscoelastic properties, and it can be adjusted to be injectable into the root canal using a syringe, then recovers the original stiffness in situ. Investigating the regenerative potential of this variant, MDP, cellularized by incorporating DPSCs and functionalized with GFs relevant to vascularization and DPSC differentiation, including TGF-β1, FGF-2 and VEGF, via heparin binding, revealed promising results [1]. VEGF has long been identified as a key stimulator of endothelial cell migration and proliferation [305]. TGF-β1 is recognized as a regulator of DP proliferation, migration and ECM production [163], while FGF-2 is able to induce cell growth and migration without affecting cytodifferentiation [306]. These modified cellularized hydrogels, when implanted into the backs of immunocompromised mice, resulted in the formation of vascularized soft connective tissues similar to the dental pulp. The incorporated GFs were able to attract host cells into the tooth cylinders, in addition to supporting cell proliferation and differentiation when seeded with DPSCs [1]. SHED was further cultured on an MPD hydrogel to study the effect of hydrogen peroxide-induced hypoxic conditions on SHED secretome. Hypoxia-challenged SHED was able to secrete anti-inflammatory secretome into the cell culture. MPD was decellularized and lyophilized to produce a biomaterial containing anti-inflammatory bioactive molecules, in order to reduce pulpal inflammation and to promote dentin pulp complex regeneration [75].

The effect of the pretreatment of the tooth cylinders with NaOCl, or with NaOCl followed by EDTA, prior to implantation of the above mentioned variant of MDP incorporating GFs and DPSCs [1] was investigated by subcutaneous implantation in the dorsal surface of mice [20]. In both groups, the implanted peptide hydrogel was degraded and replaced by a vascularized soft connective tissue similar to the dental pulp. However, noticeable differences could be observed at the cell–dentin interface where, samples pretreated with NaOCl showed resorption lacunae created by multinucleated cells with clastic activity, while following conditioning with EDTA, DPSCs formed an intimate association with the dentin surface, differentiated into odontoblasts-like cells expressing DSP, with cytoplasmic processes extending into the dentinal tubules [20]. Thus, for the optimization of any regenerative procedure, dentin preconditioning is a critical step that could greatly influence cellular differentiation and interaction at the dentin interface, which could impact the outcome of the REP.

Collectively, it could be concluded that self-assembled peptide hydrogels could act as injectable scaffolds for stem cell-based regenerative endodontics. Attaining physical and mechanical properties sufficient for clinical usage, besides ensuring their ability to attain full odontoblastic differentiation and tubular dentin formation clinically, are crucial prior to their clinical translation.

#### 4.2.6. VitroGel 3D

VitroGel is a commercially available, xenogeneic-free, synthetic polysaccharide injectable hydrogel. VitroGel 3D is biocompatible and forms a stable gel at room temperature, and its properties can be controlled through varying the hydrogel concentration. It forms a highly porous 3D culturing system, which mimics the cellular natural environment and promotes cellular interaction [73]. The SDF-1α and BMP-2 treatment of SCAP suspended in VitroGel 3D hydrogel solution effectively upregulated the early odontogenic marker RUNX-2, in addition to late odontogenic markers DMP-1, DSPP and OCN, in vitro, and these were associated with the formation of mineralized tissue surrounded by vascularized tissues in vivo following ectopic subcutaneous injection in immune-compromised mice [73]. 

Studies discussing natural hydrogels used for dentin-pulp complex regeneration are summarized in Table 2.

### 4.3. Hybrid Hydrogels

Based on the polymer’s nature when involved in hydrogel synthesis, hybrid hydrogels incorporate synthetic and natural polymeric chains, combining their characteristics and advantages and alleviating their drawbacks. Synthetic polymers provide ease of manufacturing on a large scale, and could be mechanically tuned and processed into the desired shapes at a molecular level by the polymerization and crosslinking, while natural polymers evoke an active cellular response, and provide biological recognition and cell-triggered remodeling [307]. Hybrid hydrogels are mainly composed of proteins and peptides enclosed into synthetic polymers via conjugation or polymerization, which make them compatible for in vitro studies and in vivo applications [308]. Hybrid hydrogels have become a direct approach to creating bioactive hydrogel scaffolds for dentin–pulp tissue engineering [307,309]. They can enhance cell adhesion, organization, and cell–cell interactions, which allows the fabrication of tissue constructs with improved cellular organization, mechanical integrity and electroactivity [310,311]. Hybrid hydrogel polymers involved in dentin–pulp regeneration include alginate/laponite hydrogel [56] as well as PEG-modified natural polymers, such as heparin, dextran, HA, fibrinogen and albumin [262].

#### 4.3.1. Alginate/Laponite Hydrogel

RGD alginate is a commercially available alginate modified through the incorporation of the RGD cell-binding sequence. Alginate/laponite hybrid injectable hydrogel microspheres, formed of RGD alginate and nano-silicate laponite, were successfully used as a carrier for VEGF and DPSCs. The resulting hydrogel was biocompatible, biodegradable, and supported DPSCs odontogenic differentiation and the expression of ALP, DMP-1, and Col-I in vitro. Injection of the alginate/laponite hydrogel loaded with DPSCs and VEGF into root segments followed by subcutaneous ectopic implantation in nude mice resulted in the formation of vascularized pulp-like tissues with a cell layer lining the dentin walls in vivo. The incorporation of laponite allowed the tunability of the hydrogel’s mechanical property and VEGF release [56].

#### 4.3.2. PEG-Modified Natural Polymers

PEG was used in conjugation with natural polymers such as alginate [312], fibrinogen [313], fibrin [66] HA and gelatin [314] for potential dentin–pulp complex regeneration, with promising results. PEG alginate hybrid hydrogel was evaluated for the local delivery of DPSCs and FGF for pulp regeneration in vitro and in vivo. A stereolithography 3D printed stem cell-responsive PEG diacrylate and sodium alginate composite hydrogel system (PEGDA/SA), constructed at different mass ratios of PEGDA to SA (25:1, 20:1 and 15:1), was loaded with DPSCs and basic FGF (PEGDA/SA-bFGF). The DPSCs-loaded PEGDA/SA-bFGF hydrogels exhibited high cell compatibility and supported cell proliferation, irrespective of their concentration ratios in vitro. The hydrogel system also successfully formed a well-organized pulp structure in vivo after four weeks of implantation in an immunodeficient mice model, when used at ratios of 20:1 and 15:1. The DPSCs-loaded PEGDA/SA construct without FGF addition, on the other hand, failed to form a pulp structure, while a partial loose connective tissue formation was observed with the mass ratio of 25:1 [312].

The PEG fibrinogen (PF) hydrogel composed of PEG side chains conjugated to a bioactive fibrinogen backbone was also investigated for potential dentin–pulp complex regeneration. PF is an injectable hydrogel characterized by having a tunable mechanical property which can be adjusted through controlling the degree of crosslinker concentration. PF hydrogels were cytocompatible and supported DPSCs odontogenic differentiation and mineralization. DPSCs’ morphology, odontogenic genes expression (Col I, DSPP, DMP-1, OC) and mineralization potential were strongly dependent on crosslinker concentration (0, 0.5, 1.5, and 2.5% *w*/*v*) and matrix stiffness. The highest odontogenic gene expression and mineralization potential, with lesser proliferation potential, was recorded with the hydrogel with higher crosslinker concentration [313].

The compatibility of PEGylated fibrin scaffold was examined on dental stem/progenitor cells SHED, DPSCs and PDLSCs. Dental stem/progenitor cells were cultured on PEGylated scaffolds and subjected to osteogenic induction for four weeks. An upregulation in ALP activity and osteoblast and odontoblast differentiation genes, including Col I, Col III, MMP‑2, BSP, OCN and RUNX 2, were observed. The gene expression profile was variable between different cell types, whereby SHED and PDLSCs exhibited higher expressions of collagen, while DPSCs and PDLSCs expressed higher levels of late markers of differentiation as compared to SHED, which reflected the immaturity of the SHED population, while dentin-specific markers (DSPP and DMP‑1) increased in the pulp-derived stem/progenitor cells (SHED and DPSCs). Further, SHED displayed a slightly higher proliferation rate upon culturing on either PEGylated fibrin or Col I scaffolds, as compared to Puramatrix™. Histologic analysis for in vitro cultures revealed the degradation of fibrin, the production of a collagenous matrix, and mineral deposition. SHED was selected to be loaded onto a PEGylated fibrin scaffold, incorporated in dentin disks and then implanted in immunocompromised mice. After five weeks of in vivo transplantation, the fibrin matrix was degraded and replaced by a vascularized soft connective tissue similar to dental pulp [66]. Therefore, PEGylated fibrin appeared to be a promising biomaterial for pulp tissue regeneration, since it supported cell encapsulation and promoted the growth and differentiation of dental stem cells, and can be inserted into tiny defects.

HyStem-C is a commercially available injectable hydrogel comprised of HA crosslinked with synthetic PEGDA and thiol-reactive gelatin. HyStem-C has tunable mechanical properties through changing the concentration of its components to mimic the desired tissue criteria, which allows for promoting cell viability and spreading [315]. Accordingly, HyStem-C’s components were altered to improve its mechanical strength, DPSCs biocompatibility and injectability into the root canal [314]. Firstly, PEGDA were modified by an added disulfide bond, giving rise to PEGSSDA to enable non-enzymatic cell recovery. Secondly, variable volume ratios of thiolated HA:thiolated gelatin (1:0, 3:1, 1:1, and 1:3) were utilized corresponding to HA:gelatin ratios (100:0 to 25:75) in order to encourage cell spreading. Fibronectin was subsequently added in 1:1 (*v*/*v*) ratios of thiolated HA and gelatin solutions, to examine the effects of ECM proteins on human DPSCs proliferation and migration. Finally, PEGDA 3400 (2.0% *w*/*v*) was added to the HA–gelatin–Fn mixture in a 1:4 volume ratio. Interestingly, the hydrogel gelation time decreased as the PEGSSDA crosslinker concentration increased. The PEGSSDA–HA–gelatin was biocompatible with human DPSCs, as increasing the ratios of HA:gelatin enhanced cell viability for 14 days. Additionally, cell proliferation with added fibronectin increased considerably with time in PEGDA–HA–gelatin hydrogels, while cell spreading increased at fibronectin concentrations of 0.1 μg/mL. The cell adhesion signaling cascades mediated by fibronectin binding seemed to allow cells to attach to the ECM and function well. Human DPSCs proliferation increased with increased fibronectin concentrations. Although the human DPSCs embedded in the PEGSSDA–HA–gelatin hydrogels appeared to spread early, the migration potential seemed to decrease after four days, which proved the key role of fibronectin in DPSCs spreading [314]. Both collagen and fibronectin are expressed in native dental pulp, but the mutual integrin-binding sites’ effects on human DPSCs fate in injectable hybrid scaffolds need further clarification. Studies discussing natural hydrogels used for dentin-pulp complex regeneration are summarized in Table 3. 

## 5. Comparing Natural and Synthetic Classes of Hydrogels

Natural hydrogels are biocompatible, biodegradable and bioactive, can mimic cells’ ECM, and promote cell adhesion, proliferation and differentiation, besides being not associated with sever inflammatory responses [316,317]. Natural hydrogels are also characterized by having bioactive molecules which can promote cell proliferation and function [318] in addition to their innate content of cell adhesion molecules, which can promote cellular adhesion and migration [319]. However, natural hydrogels can carry the risk of disease transmission and immune reaction, and may display batch to batch variations [7,320,321,322] in addition to their poor mechanical properties [322]. Synthetic hydrogels were developed, aiming to overcome natural hydrogel’s limitations. They are characterized by ease of standardization, large-scale production, and tunable mechanical properties, degradation rate and microstructure. They also carry no risks for disease transmission [7,321,322]. They also have a higher water retention capacity, in addition to longer shelf-life [323]. Unfortunately, synthetic hydrogels are not biologically active, and often require the incorporation of biologically active molecules and cell-binding sequences to improve cellular response [7,324].

Different synthetic and natural hydrogels were tested for their suitability in engineering dental pulp. Two classes of synthetic materials, PEG and self-assembled peptides, in addition to two natural hydrogel materials, fibrin and Col I, were tested for their biocompatibility with primary human DPSCs [325]. Three different PEG-based materials were developed—a chemically curing hydrogel (PEGchem), a light-curing hydrogel (PEGlight), and a biomimetic hydrogel (PEGbio) that was functionalized with a cell adhesion motif (Arg-Gly-Asp-Cys; PEG5k-RGDC-acrylamide) in addition to an MMP-2 sensitive enzyme-cleavable site. Two types of self-assembled peptides have also been used—a custom made self-assembling peptide, with a defined stiffness and a modified mode of gelation with a cell adhesion motif and an MMP-2-cleavable site (SAPbio), and a commercially available self-assembling peptide (Puramatrix™) (SAPcom). The primary DPSCs cultures were established from extracted third molar tooth germs validated through being STRO-1-positive, in addition to being susceptible to adipogenesis, osteogenesis and chondrogenesis differentiation. The cell viability of the DPSCs incorporated into test materials was assessed using MTT assays over two weeks. Four out of the seven materials were chosen for an in vivo model for dental pulp regeneration: fibrin, PEGchem, PEGbio and SAPbio. Selected test materials were loaded with dentin matrix proteins that had been filtrated to attain a 500 pg/mL concentration of TGF-β1, then seeded with cells, injected into human tooth roots and finally transplanted subcutaneously into immunocompromised mice for four weeks. Distinct differences were revealed between different types of scaffolds, where in vitro cell viability was considerably higher in natural materials as compared to synthetic ones. Furthermore, significant differences regarding scaffold degradation, odontoblast-like cell differentiation, tissue formation and vascularization were revealed in vivo [325]. These data provide evidence that natural materials, especially fibrin, appeared ultimately suitable for pulp tissue regeneration, regarding the efficient enhancement of odontoblastic differentiation at the cell–dentin interface. Study highlighting and comparing natural and synthetic hydrogels used for dentin-pulp complex regeneration are summarized in Table 4.

## 6. Clinical Application of Different Hydrogels for Dentin–Pulp Complex Regeneration

The successful clinical translation of a hydrogels scaffold for dentin–pulp complex regeneration has been reported. Gelatin hydrogel loaded with FGF was used for apexogenesis of immature, non-vital, permanent maxillary anterior teeth in human patients. Following access cavity and root canal mechanical preparation, intracanal bleeding was induced through over instrumentation; this was followed by injecting the hydrogel into the root canal. Follow-up radiographs revealed an increase in root length and width with a decrease in apical diameter, confirming continuation of root development [85]. Polylactic‑polyglycolic acid (PLGA) and PEG co-polymer-based hydrogel were also successfully used for the apexogenesis of the lower left second premolar with immature apex and thin radicular dentinal walls in a 20-year-old patient. The access cavity was prepared and the root canal was irrigated and dried. PEG‑PLGA hydrogel loaded with autogenous SCAP was injected into the canal, and the access cavity was sealed with a restoration. Three months post-operation, the tooth was asymptomatic, and radiographic examination revealed apical closure and thickening of the dentinal walls [260]. Furthermore, an injectable mixture combining human-treated dentin matrix and alginate was used as a pulp-capping agent for dentin regeneration clinically. This scaffold showed no difference compared with Biodentin and the mineral trioxide aggregate when used as direct pulp capping materials clinically and histologically. However, there was a difference in the thickness and quality of the hard tissue formed on the exposed pulps with human-treated dentin matrix, showing a positive tendency for dentin regeneration and conservation of pulp vitality [248].

Platelet-rich fibrin (PRF) is considered to be a natural multifunctional hydrogel with promising application in tissue regeneration [326]. Fibrin gel is formed as plasma fibrinogen, and is activated by protease thrombin during the clotting process to polymerize into a 3D network of branching fibers, giving rise to hydrogels with high elastic moduli [327]. Autologous PRF is prepared through the centrifugation of patients’ blood. It allows the local delivery of growth factors, including PDGF, VEGF, TGF-β, BMP and cells, including platelets and leukocytes, which promote tissue healing and regeneration [327]. A case study reported successful pulp regeneration with DPSCs-loaded PRF in a mature permanent tooth. Autologous DPSCs isolated from extirpated autologous inflamed dental pulp were loaded on autologous PRF in a lower premolar tooth with irreversible pulpitis, following access cavity and mechanical preparation of the root canal. The tooth was asymptomatic during follow up visits, with no signs of pulpal inflammation; pulp testing confirmed the presence of vital dental pulp and radiographic evaluation revealed no periapical pathosis [249]. A randomized controlled trial further evaluated the efficacy of PRF for the apexogenesis of necrotic permanent maxillary incisors with immature roots. Patients recruited in the study with necrotic permanent maxillary incisors were subjected to access cavity preparation, then roots were mechanically prepared, followed by the intracanal application of autologous PRF prepared by centrifugation of the patients’ blood in the absence of anticoagulants. Follow-up radiographs demonstrated periapical healing, apical closure, root lengthening and thickening of dentinal wall. The regeneration potential of PRF was superior to that of the placentrex and chitosan scaffolds [250].

## 7. Physical and Chemical Properties of the Hydrogel that Affect Cellular Behavior in Dentin–Pulp Regeneration

For REP, the physical, chemical and structural properties of the polymer are of paramount importance in dictating the cellular behavior, and hence the final biological outcome.

### 7.1. Bioactivity of the Polymer

The utilization of naturally occurring matrices, such as decellularized dentin matrix, pECM or naturally derived polymers, is a promising approach. They are bioactive, and preserve the natural cell-adhesive motifs (RGD) and MMP-binding sites that are important for cellular interaction and enhancing cell viability, proliferation, and differentiation [81,92]. In decellularized matrix hydrogel derived from human dental pulp, the protein components of this decellularized biomaterial were found to be responsible for the regulation of cellular behaviors, such as cell adhesion, proliferation and migration [247]. Optimizing the wettability of hydrophobic polymers is essential in order to enhance cell adhesion. Altering the water contact angle of the PDMS material surface from 110° to 65° was employed to optimize cell attachment [328].

### 7.2. Hydrogel Forming Ability

The hydrogel-forming properties of the employed polymer are crucial. The gelation process should be carried out using non-toxic solvents and under physiological conditions (in terms of pH and temperature), to allow easy cell encapsulation and entrapment without negatively impacting cell viability. Such hydrogel formulations offer great potential for cell and/or drug delivery.

### 7.3. Concentration of the Monomer

Tailoring the polymeric hydrogels’ mechanical properties can be done by varying different parameters, such as the polymer molecular weight, source, concentration and chemical modifications, and/or the type and degree of crosslinking [159].

Varying the monomer concentration and oligomer:monomer ratio allows for tuning the mechanical properties to cover a range of stiffnesses to match various tissues, which modulate cell–matrix interactions and could specify stem cell lineage. The stiffness and microstructure of collagen matrices could be tailored by modulating collagen concentration and ratio [61,101]. The stiffness of the matrix also affects the rate of cell proliferation, whereby cell proliferation was shown to be higher on the compliant matrix, as compared to the stiffer matrix. This might be explained by the longer time taken by the cells to penetrate the stiffer matrices [61]. Stiffness also affect cytoskeletal organization and cell shape; stiffer collagen matrices (800 Pa) decreased the cell spreading and actin fiber organization, while compliant collagen (235 Pa) supported the growth of elongated DPSCs [61]. Moreover, GelMA hydrogels of 10 and 15% (*w*/*v*) concentrations had smaller pore sizes than 5% GelMA, thus indicating the formation of denser crosslinked networks in hydrogels with higher concentrations [68].

In PDMS substrates, the stiffness of substrates increased with reducing the liquid oligomeric base to agent-curing proportions [263]. The mechanical properties of the hydrogel influence the cell morphology, which subsequently influences cell function. Increased substrate stiffness enhances DPSCs osteoblastic/odontogenic differentiation and proliferation, as evident by the increased expression of marker genes and proteins related to the odontogenic differentiation of DPSCs. The potential mechanism of stiffness-dependent osteogenic/odontogenic differentiation and proliferation might be linked to the canonical Wnt pathway [263].

### 7.4. Concentration of the Crosslinker

Controlling the crosslinker concentration and crosslinking density allows for tailoring the mechanical properties [96]. The enhanced physical properties induced via crosslinking improve the mechanical properties, such as stiffness. Modulating substrate stiffness exerts biomechanical regulatory forces, where stiffer matrices constrain the movement of tissues and cells, slow cellular movement, and the cells exert higher traction forces on their substrates, which are dissipated within the cell, and might change the protein conformation connecting the cytoskeleton and matrix [97,98]. Thus, increased stiffness and compressive strength could impact hDPCs count and odontogenic differentiation [60]. In 1,4-butanediol diglycidyl ether (BDDE)-crosslinked HA gels (HAGs), the higher the concentration of BDDE, the more rigid the hydrogel, as more crosslinking points are yielded. The higher the rigidity of the hydrogel the lower the swelling ratio and the higher the G’ and G’’ value [193]. Increasing the crosslinking density results in a reduction in pore size, while a higher BDDE concentration also resulted in the lesser degree of interconnectivity [193]. In HA/cellulose nanocrystals/platelet lysate (CNC/PL), the incorporation of a-CNCs in the HA matrix led to stiffer hydrogels and induced a reinforcement effect by acting as effective junction elements [194]. CNCs incorporation increased the degree of crosslinking, and consequently decreased the degradation rate and swelling profiles. Furthermore, CNCs incorporation resulted in a slower and longer release of the GFs by prolonging the entrapment time of the GFs [194].

Increasing the concentration of the PEGDA crosslinker in the PF hydrogels resulted in increasing the mean shear storage modulus. Furthermore, the gel point of the hydrogels ranged from 11.4 to 23.4 s with increasing amounts of PEGDA. The addition of PEGDA crosslinker significantly decreased the swelling ratio from 42 ± 7.5 in PF-0 to 29.3 ± 0.8 in PF-2.5 hydrogel. These results indicate that the increase in PEGDA crosslinker allows for a higher degree of PF hydrogel crosslinking, which results in higher modulus but also requires longer times for completion of the crosslinking process. These results indicate that an inverse correlation exists between the amount of additional PEGDA and the swelling ratio, whereby hydrogels with higher PEGDA concentration induce less swelling due to the higher network crosslinking degree with a smaller mesh size [313].

HA/CNC/PL hydrogels were produced through hydrazone crosslinking chemistry between hydrazide/amine and aldehyde groups. The CNCs content in injectable HA/CNC/PL hydrogel showed a positive effect on the gelation time of the hydrogels; the decrease in gelation time was proportional to the amount of the available aldehyde moieties for crosslinking incorporated through a-CNCs [194]. A shortened setting time and faster conversion into the gel state has been shown to enhance cellular adhesion, as compared to the sol state [60].

### 7.5. Polymer Degradability

Varying the polymer molecular weight, besides altering its mechanical properties, also affects the polymer degradation. The molecular weights of the HA polymers significantly affected the dynamic degradation, swelling, and rheological properties of the hydrogels, whereby the 18-kDa HA hydrogels degraded faster and lost their stiffness (G′) quicker than the 270-kD HA hydrogels. The degradation rate decreased and the storage modulus (G′) increased with the increase in the molecular weight of HA, while the G′ decreased with the degradation of the hydrogels [196].

The modification of non-degradable polymers is essential to allow cells/drug release, and allow replacement by regenerating the tissue. Alginates are non-biodegradable in mammals, due to a lack of alginase enzymes. The partial oxidation of alginate chains using sodium periodate makes them biodegradable, and promotes the hydrolysis of alginate in aqueous solutions [127,161]. A biodegradable variant of MDP was designed via incorporating the cell adhesion motif RGD and matrix metalloprotease-2 (MMP-2) enzyme-cleavable site for cell-mediated degradation [2].

### 7.6. Biofunctionalization

Some of the widely used polymers are not bioactive, such as alginates and synthetic polymers; they lack cell-adhesion motifs, which affects cellular adhesion and subsequent cellular processes. The biofunctionalization of such polymers is a common way to improve their bioactivity. As discussed earlier, the coupling of an entire protein is difficult to control and may trigger an unwanted immune response, and proteins are subjected to proteolytic degradation. Thus, the immobilization of cell recognition motifs, such as RGD, is a more common way to improve the polymer’s ability to interact with cells [2,83,94,165].

Puramatrix™ is a peptide-based synthetic hydrogel, consisting of alternative hydrophilic and hydrophobic amino acids arginine (R), alanine (A), and aspartic acid (D), resembling the RGD motif found in many extracellular matrix proteins such as fibronectin [329].

BMP, VEGF, FGF-2 and TGF are the principle morphogens that are being used frequently in conjunction with dental stem cells to induce a variety of cellular activities and induce various tissue structures, even when used at low very concentrations [1]. VEGF and FGF were shown to enhance angiogenesis and neovascularization in severed human DP [24], while BMPs are suggested to induce new dentin formation [25]. Dentin-derived BMP2 has been demonstrated to play a key role in inducing the differentiation of dental stem cells into odontoblasts [330]. Growth factors might be added to the matrix to supplement the biophysical factor-induced differentiation of DPSCs [24]. VEGF/chitosan/β-glycerophosphate (CS/β-GP) hydrogel was able to provide the sustained release of VEGF into the surroundings, which promoted the activity and odontogenic differentiation of DPSCs [180].

### 7.7. Rheological Properties

The polymeric hydrogel’s injectability is greatly influenced by the hydrogel viscosity evaluated by storage modulus (G′) and loss modulus (G′′). Indeed, a relatively low viscosity of the formulation is necessary to allow for the easy injection into an emptied endodontic space with a millimeter-range diameter [123]. The viscosity of the polymer solution increases with polymer molecular weight, and the polymer formulation must remain sufficiently fluid to preserve the injectability of the final formulations [123]. In 3D bioprinting, the printability of cell-laden hydrogel bioinks depends mainly on the material’s rheological properties, especially prior to crosslinking. The rheological properties of importance include the material’s viscosity and shear-thinning properties (or decreases in viscosity as a function of shear rate) [168]. In addition, DPSCs morphology and stemness were shown to be strongly correlated with the rheological properties of the 2-Aminoethyl methacrylate (AEMA-HA) hydrogels. When the hydrogels’ storage modulus was higher than 486 Pa, the DPSCs had a spherical morphology and increased stemness [196]. An appropriate “setting/gelation time” was deemed necessary; not too fast so as to enable the accurate injection of the formulation into the endodontic space, and not too long to allow for the rapid implementation of the crown filling [123]. In GC-TRS hydrogel scaffold, the glycol chitin gelation rate was very high when the temperature was raised above the body temperature, and exhibited fast sol–gel transition kinetics in vivo, which is highly desirable for injectable hydrogel scaffolds [179]. Crosslinking affected the rheological properties of the polymeric hydrogels; GelMA exhibited a ~2 kPa G′ before UV crosslinking and ~4 kPa after UV crosslinking [65]. Both G′ and G′′ increased as a function of time after raising the temperature of the buffered solution from 15 C to 37 °C [245,246].

### 7.8. Network Structure

Three-dimensional matrices recover the cell–cell cross talks and cell–matrix interactions existing in vivo in the in vitro culture environment [104,331]. The scanning electron microscopic characterization of the decellularized matrix hydrogel derived from human dental pulp exhibited an ECM-like nanofibrous structure [247]. MDP, a self-assembling peptide, self-assembles into nanofibers 6 nm in diameter, which mimic the nanoscale architecture of the natural ECM, enhancing cell-binding to the fibers via adhesion molecules and cellular interaction in all dimensions [304]. Puramatrix™ is another synthetic peptide hydrogel, which in the presence of physiological buffer spontaneously organizes into a membranous 3D hydrogel that exhibits a nano-fibrous structure with an average pore size of 50–200 nm [329]. Lyophilized chitosan hydrogels exhibited a spongy and porous microstructure, with an average pore diameter range of 100 to 200 μm, which allowed DPSC adhesion with the embedded cellular synapses in the hydrogels [180]. Crosslinked silated hydroxypropylmethylcellulose (Si-HPMC) hydrogel revealed a nano-porous macromolecular structure with an average pore diameter of 10 nm, which is greater than the diameter of the proteins, allowing the liberation of proteins and growth factors [226].

3D bioprinting, a relatively novel rapid prototyping technology, allows for precise control of the external architecture and internal microstructure of the scaffolds, and the scaffold’s pore interconnectivity and pore diameter. Such printed matrices attempt to mimic the in vivo cellular environment for improved cellular interactions and desirable biological response [168,169].

Incorporating bioactive glass nano-particles into thermo-responsive injectable hydrogels also provided a large surface area with highly charged surfaces, so as to increase bioactivity, accompanied by the highly porous structure of the scaffold. This modification offers a large surface area and interconnected pores for the ingrowth of the cells [228].

### 7.9. pH of the Polymer Solution

The pH of the employed polymer solution must be close to neutrality to avoid detrimental effects on cell viability. For preparing chitosan-enriched fibrin hydrogel, the pH of the solution after the acidic dissolution of chitosan in deionized water must be increased. Following testing several batches of chitosan with 20%, 33% and 40% DA, only the solution of chitosan with 40% DA could be brought to pH neutrality without forming precipitates [123]. The stock solution of Puramatrix™ (1% *w*/*v*) exhibits pH 3.0, which can adversely affect cell viability. Therefore, it is important to work quickly to minimize the amount of time that cells are in contact with this material prior to the addition of culture medium [329].

## 8. Conclusions

Despite the fact that dentin–pulp tissue regeneration in laboratory conditions provided functional odontoblasts capable of regenerating tubular dentin, the clinical translation of these experiments will require cooperation between biologists, biomaterials scientists and clinicians in order to develop safe and effective approaches to dental pulp regeneration. Among the obstacles to overcome prior to clinical translation is the need for exogenous chemotactic factors that have to be incorporated into the scaffolds for the active recruitment of cells from the periapical region in cell-free tissue-engineering approaches [332], yet the plethora of GFs included and expressed by dentin makes this task very complex. Interestingly, the biological ligand on a scaffold surface is a major factor that controls both cell attachment and spreading. Despite the stated hypothesis that synthetic materials might be better candidates for tissue engineering approaches, given the high control over their properties and being functionalized with specific bioactive motifs, it seems that natural biomaterials, particularly collagen and fibrin, are superior to synthetic materials in dental pulp tissue regeneration. Still, even though hydrogels show great potential in fulfilling the criteria of the ideal scaffold material for dentin–pulp complex regeneration, devising an ideal scaffold material that is injectable, biodegradable, biocompatible, non-immunogenic. characterizied by high porosity and adequate mechanical properties, which is also biomimetic, can act as a 3D matrix to mimic cells’ natural habitats. In addition to being easily biofunctionalized and combined with stem/progenitor cells and can support cellular adhesion, migration, proliferation and differentiation, is a task yet to be accomplished.

## Figures and Tables

**Table 1:** Natural hydrogels.

	Natural Hydrogels						
Author	Hydrogel Used	Type of Study	Hydrogel Modification	Hydrogel Properties	Cells Used	Upregulated Biological Molecules	Outcomes
	**Collagen Hydrogel**						
Souron et al., 2014 [104]	Collagen	In vivo		3D collagen matrices, mimic in vivo cell–cell and cell–matrix interactions, and regulate cell growth	Rat pulp cells labeled with indium-111-oxine		1 month following implantation, active fibroblasts, new blood vessels and nervous fibers were present in the cellularized 3D collagen hydrogel.
Kwon et al., 2017 [60]	Collagen	In vitro	Crosslinked with cinnamaldehyde (CA).	Crosslinked collagen with shorter gelation time enhances cellular adhesion. Higher stiffness enhances odontogenic differentiation.	Human dental pulp stem cells (DPSCs)	Dentin sialophosphoprotein (DSPP), Dentin matrix protein 1 (DMP-1), Matrix extracellular phosphoglycoprotein (MEPE), Osteonectin (ON)	CA shortened the setting time, increased compressive strength and surface roughness of collagen hydrogels. CA-crosslinked hydrogels promoted the proliferation and odontogenic differentiation of human DPSCs
Pankajakshan et al., 2020 [61]	Collagen	In vitro	Varying hydrogel stiffnesses through varying oligomer concentrations. Incorporation of Vascular endothelial growth factor (VEGF) into 235 Pa collagen or Bone morphogenetic protein 2 (BMP-2) into the 800 Pa ones.	Stiffness affect cytoskeletal organization and cell shape and specify stem cell lineage.	DPSCs	von Willebrand Factor (vWF), platelet endothelial cell adhesion molecule 1 (PECAM-1), vascular endothelial-cadherin	Collagen hydrogels with tunable stiffness supported cell survival, and favored differentiation of cells to a specific lineage. DPSCs cultured in 235 Pa matrices showed an increased expression of endothelial markers, cells cultured in 800 Pa showed increased alkaline phosphatase (ALP) activity and Alizarin S staining.
	**Gelatin Hydrogel**						
Kikuchi et al., 2007 [84]	Gelatin hydrogel	In vivo	Crosslinked gelatin hydrogel microspheres were impregnated with fibroblast growth factor 2 (FGF-2) and mixed with collagen sponge pieces.	Gelatin hydrogel microspheres (the water content is 95 vol%; diameters ranged from 5–15 µm; the average of diameter was 10 µm)		DSPP	Controlled release of FGF2 from gelatin hydrogels induced the formation of dentin-like particles with dentin defects above exposed pulp.
Ishimatsu et al., 2009 [117]	Gelatin hydrogel	In vivo	Gelatin hydrogel microspheres incorporating FGF-2.	Gelatin hydrogel microspheres (the water content is 95 vol%, the average diameter was 10 µm)		DMP-1	Dentin regeneration on amputated pulp can be regulated by adjusting the dosage of FGF-2 incorporated in biodegradable gelatin hydrogels.
Nageh et al., 2013 [85]	Gelatin hydrogel	Clinical trial	FGF incorporated in gelatin hydrogel.	Acidic gelatin hydrogel microspheres with a mean diameter of 59 µm and 95.2% water content.			Follow-up X-ray revealed an increase in root length and width with a reduction in apical diameter confirming the root’s development.
Bhatnagar et al., 2015 [62]	Gelatin hydrogel	In vitro	enzymatically crosslinked with microbial transglutaminase (mTG).	Hard and soft gelatin–mTG gels consist of 1.125 mL and 1.488 mL of a 10% gelatin solution with 0.375 mL (3:1 (*v*/*v*) gelatin: mTG), and 0.012 mL (125:1 (*v*/*v*) gelatin: mTG) of mTG respectively	DPSCs	Osteocalcin (OCN), ALP, DSPP	Enzymatically crosslinked gelatin hydrogels are a potential effective scaffold for dentin regeneration regardless of matrix stiffness or chemical stimulation using dexamethasone.
Miyazawa et al., 2015 [63]	Gelatin hydrogel	In vitro In vivo	Simvastatin-lactic acid grafted gelatin micelles, mixed with gelatin, followed by chemical crosslinking to form gelatin hydrogels.	Carboxymethylcellulose (CMC) value of micelles was 79 μg/mL. Water solubilization of Simvastatin 43 wt.%. Simvastatin in the gelatin 3.23 wt.%. The sizes of granules 500 μm with rough surfaces and uniformly sized pores	DPSCs	ALP, Dentin sialoprotein (DSP), BMP-2	It is possible to achieve odontoblastic differentiation of DPSCs through the controlled release of Simvastatin from gelatin hydrogel.
	**Gelatin Methacrylate Hydrogel**						
Athirasala et al., 2017 [68]	Gelatin Methacrylate hydrogel (GelMA)	In vitro	Gelatin with methacrylic anhydride.	GelMA hydrogels of 5, 10 and 15% (*w*/*v*) concentrations showed a honeycomb-like structure. Both 10% and 15% hydrogel groups appeared to have smaller pore sizes than 5% GelMA.	Odontoblasts like cells (OD21) and endothelial colony- forming cells		Pre-vascularized hydrogel scaffolds with microchannels fabricated using GelMA is a simple and effective strategy for dentin–pulp complex regeneration.
Khayat et al., 2017 [64]	GelMA	In vivo	Gelatin with methacrylic anhydride.		DPSCs and human umbilical vein endothelial cells (HUVECs)		GelMA hydrogel combined with human DPSC/human umbilical vein endothelial cells as promising pulpal revascularization treatment to regenerate human dental pulp tissues.
Ha et al., 2020 [72]	GelMA	In vitro	Gelatin with methacrylic anhydride hydrogels of increasing concentrations.	Increasing polymer concentrations from 5% to 10% and 15% (*w*/*v*), resulting in increasing extents of crosslinking. The elastic moduli of hydrogels, increased with increase in polymer concentration from 1.7 kPa for 5% GelMA to 7 kPa and 16.4 kPa for 10% and 15% GelMA hydrogels, respectively.	stem cells of the apical papilla (SCAP)		Substrate mechanics and geometry have a statistically significant influence on SCAP response.
Park et al., 2020 [65]	GelMA	In vitro	GelMA conjugated with synthetic BMP-2 mimetic peptide prepared into bioink.	GelMA exhibited a ~2 kPa storage modulus (G’) before crosslinking and ~4 kPa after crosslinking.	DPSCs	DSPP, OCN	BMP peptide-tethering bioink could accelerate the differentiation of human DPSCs in 3D bioprinted dental constructs.
Jang et al., 2020 [67]	GelMA	In vitro In vivo	Thrombin solution added to GelMA hydrogel.		DPSCs		Gelatin hemostatic hydrogels may serve as a viable regenerative scaffold for pulp regeneration.
	**Fibrin Hydrogel**						
Meza 2019 [249]	Platelet Rich Fibrin (PRF)	Case report			DPSCs		Autologous DPSCs isolated from extirpated autologous inflamed dental pulp were loaded on autologous PRF in lower premolar tooth with irreversible pulpitis for successful regeneration.
Ducret et al., 2019 [123]	Fibrin–chitosan	In vitro	Enriching the fibrin-hydrogel with chitosan.	10 mg/mL fibrinogen and 0.5% (*w*/*w*), 40% DA chitosan, formed a hydrogel at physiological pH (≈7.2), which was sufficiently fluid to preserve its injectability without affecting fibrin biocompatibility	DPSCs		Chitosan imparted antibacterial activity to fibrin hydrogel, reducing *E. faecalis* growth. The blending of chitosan in fibrin hydrogels did not affect the viability, proliferation and collagen-forming capacity of encapsulated DPSCs as compared to unmodified fibrin.
Mittal et al., 2019 [250]	PRF	Clinical trial					PRF and collagen are better scaffolds than placentrex and chitosan for apexogenesis of immature necrotic permanent teeth.
Bekhouche et al., 2020 [82]	Fibrin	In vitro	Incorporation of clindamycin loaded Poly (D, L) Lactic Acid nanoparticles (CLIN-loaded PLA NPs).	Fibrin hydrogel constituted a reservoir of CLIN-loaded PLA NPs inhibiting *E. faecalis* growth without affecting cell viability and function.	DPSCs		Fibrin hydrogels containing CLIN-loaded PLA NPs showed an antibacterial effect against *E. faecalis* and inhibited biofilm formation. DPSCs viability and type I collagen synthesis in cellularized hydrogels were similar to the unmodified groups.
Renard et al., 2020 [131]	Fibrin-chitosan	In vivo	Same formulation of fibrin–chitosan hydrogel as used by Ducret et al., 2019 [123]	40% DA chitosan incorporation in the fibrin hydrogel did not modify modify dental pulpi inflammatory/immune response and triggered polarization of pro-regenerative M2 macrophages.			In in vivo model of rat incisor pulpotomy, fibrin–chitosan hydrogels imparted a similar inflammatory response in the amputated pulp as unmodified fibrin. Both groups enhanced the polarization of pro-regenerative M2 macrophages.
Zhang et al., 2020 [71]	Fibrin	In vitro	Fibrin hydrogel loaded with DPSCs-derived extracellular vesicles (EVs).	2 mg/mL fibrinogen formed a hydrogel which was able to retain and preserve the activity of EVs. Forming the most extensive tubular network forming at an EVs concentration of 200 µg/mL	Co-culture of DPSCs and HUVECs	VEGF	Investigated hydrogels enhanced rapid neovascularization under starvation culture, increased deposition of collagen I, III, and IV, and promoted the release of VEGF.
	**Matrigel 3D**						
Mathieu et al., 2013 [143]	Matrigel	In vitro			DPSCs		Encapsulating transforming growth factor beta1 (TGF-b1) and FGF-2 in a biodegradable Poly glycolide-*co*-lactide (PGLA) microsphere
Ito et al., 2017 [77]	Matrigel	In vivo			Bone marrow mesenchymal stem cells (BMMSCs)	Nestin, DSPP	Pulp tissue regeneration was successfully achieved.
Sueyama et al., 2017 [78]	Matrigel	In vivo			BMMSCs and endothelial cells (ECs).	DSPP, Nestin Bcl-2, Cxcl1, Cxcr2, VEGF	The implantation of ECs with mesenchymal stem cells accelerated pulp tissue regeneration/healing and dentin bridge formation.
Gu et al., 2018 [79]	Matrigel	In vivo			BMMSCs		M1-to-M2 transition of macrophages plays an important role in creating a favorable microenvironment necessary for pulp tissue regeneration.
Kaneko et al., 2019 [80]	Matrigel	In vivo			BMMSCs nucleofected with pVectOZ-LacZ plasmid encoding β-galactosidase	DSPP	BMMSCs could differentiate into cells involved in mineralized tissue formation in the functionally relevant region.
	**Keratin Hydrogel**						
Sharma et al., 2016 [69]	Keratin hydrogel	In vitro		Highly branched interconnected porous micro-architecture with a maximum average pore size of 160 µm and minimum pore size of 25 µm. G′ > G″ indicates the elastic solid-like nature of the gel. After 3 months the degradation rate was 68%.	odontoblast-like cells (MDPC-23)	ALP, DMP-1	Keratin enhanced proliferation and odontoblastic differentiation of odontoblast-like cells. Keratin hydrogels may be a potential scaffold for pulp–dentin regeneration.
Sharma et al., 2016 [70]	Keratin hydrogel	In vitro In vivo		Highly branched interconnected porous micro-architecture with a maximum average pore size of 160 µm and minimum pore size of 25 µm. G′ > G′′ indicates the elastic solid-like nature of the gel. After 3 months the degradation rate was 68%.	odontoblast-like cells (MDPC-23) and DPSCs		Keratin hydrogel enhanced odontogenic differentiation of odontoblast-like cells and enhanced reparative dentin formation.
Sharma et al., 2017 [150]	Keratin hydrogel	In vivo		Branched interconnected porous micro-architecture with average pore size 163.5 and porosity 82.8%. There was a gradual increase in G’ from 7% to 20% (*w*/*v*) gel concentration. The average contact angle was 35.5°.			Keratins hydrogel can be a source for biological treatment options for dentin–pulp complex.
	**Alginate Hydrogel**						
Dobie et al., 2002 [86]	Alginate	In vitro	TGF- β1 or HCL acid-treatment of the hydrogels.	Alginate hydrogels are valuable for delivery of growth factors (GFs) (or agents to release endogenous GFs) to enhance reparative processes of dentin–pulp complex.			Alginate hydrogel acted as an efficient carrier for TGF- β1. Furthermore, acid treatment of the hydrogel aided in the release of TGF- β1 from dentin matrix. Alginate–TGF–β1 blends stimulated reactionary dentinogenic responses with increased predentin width.
Bhoj et al., 2015 [83]	Alginate	In vitro	Arginine-glycine-aspartic acid (RGD)-modified alginate hydrogels, loaded with VEGF and FGF-2.	RGD–alginate matrix acted as pulp replacement, compatible with the DPSCs and HUVECs, and can deliver VEGF and FGF-2.	Co-culture of DPSCs and HUVECs		Combined addition of FGF and VEGF led to an increased proliferation of both DPSCs and HUVECs in the hydrogels. RGD-modified alginate can efficiently retain VEGF and FGF-2.
Smith et al., 2015 [81]	Alginate	In vitro	Alginate hydrogel doped with bovine dental pulp extracellular matrix (pECM).	3D Alginate hydrogel doped with pECM formed 3D matrices. pECM provides additional signals for differentiation.	Primary dental pulp cells		Induced differentiation in the mineralizing medium resulted in time-dependent mineral deposition at the periphery of the hydrogel.
Verma et al., 2017 [127]	Alginate–fibrin	In vivo	Oxidized alginate–fibrin hydrogel microbeads.	7.5% oxidized alginate coupled with fibrinogen concentration of 0.1% enhanced microbead degradation, cell release, and proliferation.	DPSCs		Oxidized alginate–fibrin hydrogel microbeads encapsulating DPSCs showed similar regenerative potential to traditional revascularization protocol in ferret teeth. In both groups, the presence of residual bacteria affected root development.
Athirasala et al., 2018 [168]	Alginate	In vitro	Blending alginate hydrogels with soluble and insoluble fractions of the dentin matrix as a bioink for 3D printing.	Dentin matrix proteins preserve the natural cell-adhesive (RGD) and MMP-binding sites, which are lacking in unmodified alginate, that are important for viability, proliferation, and differentiation.	SCAP	ALP, Runt-related transcription factor -2 (RUNX2)	Alginate and insoluble dentin matrix (in 1:1 ratio) hydrogels bioink significantly enhanced odontogenic differentiation of SCAP under the effect of the soluble dentin molecules in the hydrogel.
Yu et al., 2019 [169]	Alginate and gelatin hydrogels	In vitro	3D bioprinted crosslinked composite alginate and gelatin hydrogels (4% and 20% by weight, respectively).	3D printing accurately controls the interconnected porosity and pore diameter of the scaffold, and imitate natural cell tissue in vivo.	DPSCs	ALP, OCN, DSPP	3D-printed alginate and gelatin hydrogels aqueous extracts are more suitable for the growth of DPSCs, and can better promote cell proliferation and differentiation.
	**Chitosan Hydrogel**						
Park et al., 2013 [179]	Chitosan hydrogel	In vitro	N-acetylation of glycol chitosan	Glycol chitosan (0.2 g) and acetic anhydride (0.87 g) were dissolved in 50 mL of a mixture of distilled water and methanol (50/50, *v*/*v*) degree of acetylation 90% pore size ranged from 5 to 40 mm.	Human DPSCs	DSPP, DMP-1, ON, osteopontin	Glycol chitin-based thermo-responsive hydrogel scaffold promoted the proliferation and odontogenic differentiation of human DPSCs.
El Ashiry et al., 2018 [182]	Chitosan hydrogel	In vivo		Chitosan 1 g; 77% deacylation, high molecular weight, was dissolved in 2% acetic acid.	DPSCs		DPSCs and GFs incorporated in chitosan hydrogel can regenerate pulp–dentin-like tissue in non-vital immature permanent teeth with apical periodontitis in dogs.
Wu et al., 2019 [180]	Chitosan hydrogel	In vitro	beta-sodium glycerophosphate added to chitosan (CS/*β*-GP).	viscosity: 200–400 m Pa·s 2% (*w*/*v*) chitosan solution 56% (*w*/*v*) beta-sodium glycerophosphate (*β*-GP) solution CS: *β*-GP is 5/L	DPSCs	VEGF, ALP, OCN, Osterix, DSPP	CS/*β*-GP hydrogel could release VEGF continually and promote odontogenic differentiation of DPSCs.
Zhu et al., 2019 [181]	Chitosan hydrogel	In vitro	Ag-doped bioactive glass micro-size powder particles added to chitosan (Ag-BG/CS).	Ag-BG/CS pore diameter reaching around 60–120 μm.	DPSCs	OCN, ALP, RUNX-2	Ag-BG/CS enhanced the odontogenic differentiation potential of lipopolysaccharide-induced inflammatory-reacted dental pulp cells and expressed antibacterial and anti-inflammatory activity.
	**Hyaluronic Acid Hydrogel**						
Chrepa et al., 2016 [198]	Hyaluronic acid (HA) hydrogel	In vitro		SCAP/Restylane 1:10 concentration SCAP/Matrigel mixture at 1:1 concentration 1,4-butanediol diglycidyl ether; DVS, divinyl sulphone crosslinking agent	SCAP	ALP, DSPP, DMP-1, MEPE gene	HA injectable hydrogel promoted SCAP survival, mineralization and differentiation into an odontoblastic phenotype.
Yang et al., 2016 [193]	Hyaluronic acid hydrogel	In vivo	HA crosslinked with 1,4-butanediol diglycidyl ether	HA (1.5 × 10^6^ Da) 1,4-Butanediol diglycidyl ether crosslinking agent HA concentration of 20 mg mL^−1^ gel particles of 0– 400 mm.	Dental mesenchymal cells		HA is an injectable scaffold that can regenerate cartilage and dentin–pulp complex.
Almeida et al., 2018 [195]	Hyaluronic acid hydrogel	In vitro	Photo crosslinking of methacrylated HA incorporated with PL.	High molecular weight (1.5–1.8 MDa) HA 1% (*w*/*v*) HA solution (in distilled water) reacted with methacrylic anhydride (10 times molar excess) methacrylated disaccharides% was 10.9 ± 1.07% HA hydrogels incorporating 100% (*v*/*v*) PL Met-HA was dissolved at a concentration of 1.5% (*w*/*v*) in both of the PBS and PL photoinitiator solutions.	Human DPSCs	ALP, collagen type I A 1 strand (COLIA1)	HA hydrogels incorporating PL increased the cellular metabolism and stimulate the mineralized matrix deposition by hDPSCs.
Silva et al., 2018 [194]	Hyaluronic acid hydrogel	In vitro Ex vivo	HA hydrogels incorporating cellulose nanocrystals and enriched with Platelet lysate (PL).	1 wt. % ADH-HA, 1 wt. % a-HA, 0.125 to 0.5 wt. % a-CNCs in 50 *v*/*v*% PL solution.	Human DPSCs		HA hydrogel enabled human DPSCs survival and migration.
Zhu et al., 2018 [197]	Hyaluronic acid hydrogel	In vivo	Crosslinked HA hydrogel	Crosslinked HA gel (24 mg/mL) mixed with cells at 1:1–1:1.4 (*v*/*v*, i.e., gel/cells) ratio with final cell concentration of *2 · 107/mL 1,4-butanediol diglycidyl ether; DVS, divinyl sulphone crosslinking agent 9%	DPSCs	Nestin, DSPP, DMP-1, Bone sialoprotein	HA hydrogel regenerated pulp-like tissue with a layer of dentin-like tissue or osteodentin along the canal walls.
Niloy et al., 2020 [196]	Hyaluronic acid hydrogel	In vitro	Converting sodium salt of HA into Tetrabutylammonium salt and subsequent conjugation of Aminoethyl methacrylate (AEMA) to HA backbone.	AEMA-HA macromers of two different molecular weights (18 kD and 270 kD) 1 g of H/100 mL deionized water, mixed with 12.5 g of ion exchange resin was converted from its hydrogen form to its TBA form AEMA hydrochloride (0.25 equivalent to HA repeat units)	DPSCs	NANOG, SOX2	HA hydrogels have great potential to mimic the in vivo 3D environment to maintain the native morphological property and stemness of DPSCs.
	**Agarose Hydrogel**						
Cao et al., 2016 [214]	Agarose hydrogel	In vitro	Calcium chloride (CaCl _2_) Agarose hydrogel	1.0 g of Agarose powder, 1.9 g of CaCl _2_ H_2_0			Agarose hydrogel promoted occlusion of dentinal tubules and formation of enamel prisms-like tissue on human dentin surface.
	**Cellulose Hydrogel**						
Teti et al., 2015 [227]	Cellulose hydrogel	In vitro	Hydroxyapatite was loaded inside CMC hydrogel	Degree of carboxymethylation of 95% (CMC) (average MW 700 KDa)	DPSCs	ALP, RUNX2, COL-IA1, SPARC, DMP-1, DSPP	CMC–hydroxyapatite hydrogel up regulated the osteogenic and odontogenic markers expression and promoted DPSCs adhesion and viability.
Aubeux et al., 2016 [226]	Cellulose hydrogel	In vitro	Silanes grafted along the hydroxy-propyl-methyl-cellulose chains.	nanoporous macromolecular structure. Pores have an average diameter of 10 nm		TGF-b1	Cellulose hydrogel enhanced non-collagenous matrix proteins release from smashed dentin powder.
Iftikhar et al., 2020 [228]	Cellulose hydrogel	In vitro		The surface area, average pore size and particle size of BAG (45S5 Bioglass^®^) were 65m2/g 5.7 nm, and 92 nm, respectively.	MC3T3-E1 cells differentiated into osteoblasts and osteocytes.		The prepared injectable bioactive glass, hydroxypropylmethyl cellulose (HPMC) and Pluronic F127 was biocompatible in an in vitro system and has the ability to regenerate dentin.
	**Extracellular Matrix Hydrogel**						
Chatzistavrou et al., 2014 [246]	Extracellular matrix (ECM) hydrogel	In vitro	silver-doped bioactive glass (Ag-BG) incorporated into ECM	Ag-BG powder form with particle size < 35μm. ECM concentration of 10 mg/mL ECM60/Ag-BG40, ECM50/Ag-BG50, ECM30/Ag-BG70 weight ratio.	DPSCs		Ag-BG/ECM presented enhanced regenerative properties and anti-bacterial action.
Wang et al., 2015 [245]	ECM hydrogel	In vivo In vitro	silver-doped bioactive glass (Ag-BG) incorporated into ECM	Ag-BG powder form with particle size < 35μm. ECM pepsin digest stock solutions of 10 mg ECM/mL (dry weight) Ag-BG: ECM = 1:1 in wt. %.	DPSCs		Ag-BG/ECM showed antibacterial property, induced dental pulp cells proliferation and differentiation. The in vivo results supported the potential use of Ag-BG/ECM as an injectable material for the restoration of lesions involving pulp injury.
Li et al., 2020 [247]	ECM hydrogel	In vitro		Pre-gel solution was diluted into 0.75% *w*/*v* and 0.25% *w*/*v*.	Human DPSCs	DSPP, DMP-1	decellularized matrix hydrogel derived from human dental pulp effectively contributed to promoting human DPSCs proliferation, migration, and induced multi-directional differentiation.
Holiel et al., 2020 [248]	ECM hydrogel	Clinical trial	Human treated dentin matrix hydrogel was dispersed in sodium alginate solution	Particle sized powder (range 350–500 μm) 5% (*w*/*v*) of sodium alginate 0.125 g of sterile human treated dentin matrix was dispersed in the sodium alginate solution with a mass ratio of 1:1			Treated dentin matrix hydrogel attained dentin regeneration and conservation of pulp vitality.

**Table 2:** Synthetic hydrogels.

	Synthetic Hydrogels						
Author	Hydrogel Used	Type of Study	Hydrogel Modification	Hydrogel Properties	Cells Used	Upregulated Biological Molecules	Outcomes
	**PLA Based Polymers**						
Shiehzadeh et al., 2014 [260]	Polylactic polyglycolic acid–polyethylene glycol (PLGA-PEG)	Clinical trial			Stem/progenitor cells from the apical dental papilla (SCAP)		Biologic approach can provide a favorable environment for clinical regeneration of dental and paradental tissues.
	**Synthetic Self-Assembling Peptide Hydrogel (Peptide Amphiphiles)**						
Galler et al., 2008 [74]	Synthetic peptide amphiphiles	In vitro	Peptide amphiphiles involves arginine–glycine–aspartic acid (RGD) and an enzyme-cleavable site	Peptide was dissolved at pH 7.0 to attain stock solution of 2% by weight	stem/progenitor cells of exfoliated deciduous teeth (SHED) and dental pulp stem cells (DPSCs)		The hydrogels are easy to handle and can be introduced into small defects, therefore this novel system might be suitable for dental tissue regeneration.
	**Multi Domain Self-Assembling Peptide (MDP) Hydrogel**						
Galler et al., 2011 [20]		In vivo	MDP functionalized with transforming growth factor (TGF)-β1, fibroblast growth factor (FGF)-2, and vascular endothelial growth factor (VEGF) via heparin binding		DPSCs		In tooth slices, implanted hydrogel degraded and replaced by a vascularized connective tissue similar to dental pulp. Pretreatment of the tooth cylinders with NoOCl showed resorption lacunae. With NaOCl followed by ethylenediaminetetraacetic acid (EDTA), DPSCs differentiated into odontoblasts-like cells intimately associated with the dentin surface.
Galler et al., 2012 [1]	MDP	In vivo	MDP functionalized with TGF-β1, FGF-2, and VEGF via heparin binding		DPSCs		Hydrogels implanted into the backs of immunocompromised mice resulted in the formation of vascularized soft connective tissue similar to dental pulp.
Colombo et al., 2020 [75]	MPD hydrogel	In vitro			SHED		Decellularized and lyophilized MDP produced a biomaterial containing anti-inflammatory bioactive molecules that can provide a tool to reduce pulpal inflammation to promote dentin–pulp complex regeneration.
	**RADA16-I Hydrogels Self-Assembling Peptide**						
Cavalcanti et al., 2013 [291]	A commercial self-assembling peptide	In vitro		0.2% Puramatrix™ (1% *w*/*v*)	DPSCs	Dentin matrix protein (DMP)-1, Dentin sialophosphoprotein (DSPP)	DPSCs expressed DMP-1 and DSPP after 21 days culturing in dentin slices containing Puramatrix^TM^. The surviving dentin provided signaling molecules to cells suspended in Puramatrix^TM^.
Rosa et al., 2013 [76]	A commercial self-assembling peptide	In vitro In vivo		0.2% Puramatrix™ (1% *w*/*v*)	SHED	DMP-I, DSPP, matrix extracellular phosphoglycoprotein (MEPE)	Upon mixing SHED with Puramatrix™ hydrogel for 7 days and injecting the construct into roots of human premolars, the cells survived and expressed (DMP-I, DSPP, MEPE) in vitro. Pulp-like tissue with odontoblasts able to form neo-dentinal tubules was observed in vivo.
Dissanayaka et al., 2015 [276]	A commercial self-assembling peptide	In vitro In vivo		Among different Puramatrix™ (1% *w*/*v*) concentrations, 0.15% was the optimal.	DPSCs and human umbilical vein endothelial cells (HUVECs)		Puramatrix^TM^ enhanced in vitro cell survival, migration and capillary formation. Co-cultured groups on Puramatrix^TM^ exhibited more extracellular matrix, mineralization and vascularization than DPSC-monocultures in vivo.
Nguyen et al., 2018 [88]	RADA16-I	In vitro	incorporation of dentonin sequence	Ribbonlike nanofibers with height (∼2 nm) and width (∼14 nm)	DPSCs		The self-assembled peptide platform holds promise for guided dentinogenesis.
Huang, 2020 [280]	RADA16-I	In vitro		Low concentration (0.125%, 0.25%) caused higher cell proliferation rate than high concentration (0.5%, 0.75%, 1%)	DPSCs and umbilical cord mesenchymal stem cells	DSPP, DMP-1, Alkaline phosphatase (ALP), osteocalcin (OCN)	The co-culture groups promoted odontoblastic differentiation, proliferation and mineralization.
Mu et al., 2020 [87]	RADA16-I	In vitro	incorporated with stem cell factor	100 ng/mL was the optimum concentration of the stem cell factor. Nanofibers and pores diameter were (10–30nm and 5–200nm, respectively)	DPSCs and HUVECs		Stem cell factor incorporate RADA16-I holds promise for guided pulp regeneration.
Zhu et al., 2019 [142]	Cells were cultured on Matrigel before being loaded on commercial self-assembling peptide	In vitro In vivo		300 μL 1% Puramatrix™ (1% *w*/*v*)	DPSCs overexpressing Stromal derived factor-(SDF)-1 and vascular endothelial growth factor (VEGF)	SDF-1, VEGF	Combination of VEGF- and SDF-1-overexpressing DPSCs cultured on Matrigel before being loaded on Puramatrix^TM^ enhanced the area of vascularized dental pulp regeneration in vivo.
Xia et al., 2020 [89]	Self-assembling peptide	In vitro In vivo	incorporation of RGD, VEGF mimetic peptide sequence	The nanofibers’ diameters of functionalized peptide were thicker than pure RAD. that the stiffness of RAD/ RGD-mimicking peptide (PRG)/ VEGF-mimicking peptide: (KLT) hydrogels was greater than the others	DPSCs and HUVECs		Modified self-assembling peptide hydrogel effectively stimulated stem cells angiogenic and odontogenic differentiation in vitro and dentin–pulp complex regeneration in vivo.
	**Poly-dimethylsiloxane Hydrogel**						
Liu et al., 2017 [263]	Poly-dimethylsiloxane (PDMS)	In vitro		Stiffness for 10:1, 20:1, 30:1 and 40:1 was 135, 54, 16 and 1.4 kPa and roughness was 55.67, 53.38, 50.95, and from 47.32 to 42.50nm. Water contact angle was 65°.	DPSCs	osteopontin (OPN), runt-related transcription factor (RUNX)-2, Bone morphogenetic protein	Osteogenic and odontogenic markers were positively correlated to the substrate stiffness. The results revealed that the mechanical properties promoted the function of DPSCs related to the Wnt/β-catenin pathway.
	**Poly-N-isopropylacrylamide Gel**						
Itoh et al., 2018 [267]	Poly-N-isopropylacrylamide (NIPAAm)	In vitro In vivo	NIPAAm crosslinked by PEG-DMA	Decrease in wet weight from 1 to 0.18 at 508 C. Change in surface area from 1 (258 C) to 0.62 (508 C) within 1 h. High wettability.	DPSCs	DSPP in the outer cell layer, Nanog in the center of the constructs	DPSCs in the outer layer of the constructs differentiated into odontoblast-like cells, while DPSCs in the inner layer maintained their stemness. Pulp-like tissues rich in blood vessels were formed in vivo.
	**Polyethylene Glycol**						
Komabayashi et al., 2013 [275]	PEG	In vitro	PEG–maleate–citrate (PEGMC) (45% *w*/*v*), acrylic acid (AA) crosslinker (5% *w*/*v*), 2,2′-Azobis (2-methylpropionamidine) dihydrochloride (AAPH) photo-initiator (0.1% *w*/*v*),	Optimum cell viability with exposure time of 30 s with a monomer and AAPH concentration of 0.088% and up to 1%, respectively	L929 cells		Cell viability remained up to 80% after 6 h. Controlled Ca2+ release was attained. The viscosity and injection ability into plastic root canal blocks were confirmed in a dental model.
	**VitroGel 3D**						
Xiao et al., 2019 [73]	Vitrogel	In vitro In vivo		VitroGel diluted with deionized water 1:2.	SCAP	RUNX-2,DMP-1, DSPP, OCN	VitroGel 3D promoted SCAP proliferation and differentiation. SDFr-1α and BMP-2 co-treatment induced odontogenic differentiation of human SCAP cultured in the VitroGel 3D in vitro and in vivo

**Table 3:** Hybrid hydrogels.

	Hybrid Hydrogels						
Author	Hydrogel Used	Type of Study	Hydrogel Modification	Hydrogel Properties	Cells Used	Upregulated Biological Molecules	Outcomes
	**Alginate/laponite hydrogel**						
Zhang et al., 2020 [56]	Alginate/laponite hydrogel	In vitro In vivo	Arginine-glycine-aspartic acid (RGD) modified alginate and nano-silicate laponite hydrogel microspheres, encapsulating human dental pulp stem cells (DPSCs) and vascular endothelial growth factor (VEGF).	The RGD-Alg had 55% degradation rate and the RGD- alginate/laponite exhibited 45% while pure alginate degraded only 20% after 28 days.	DPSCs	Alkaline phosphatase (ALP), Dentine matrix protein 1 (DMP-1), collagen I (Col-I)	Hybrid RGD modified alginate/laponite hydrogel microspheres has a promising potential in vascularized dental pulp regeneration.
	**PEG-modified natural polymers**						
Galler et al., 2011 [66]	PEGylated fibrin	In vitro In vivo	PEGylated fibrinogen with added thrombin.	Carboxylated N-hydroxysulfosuccinimide-active ester polyethylene glycol (PEG) (MW = 3400 Da) added to 40 mg/mL of bovine fibrinogen in TRIS- saline at a molar ratio of 10:1. Equal volume of thrombin (200 U/mL in 40 mM CaCl2) was added	Stem cells isolated from human exfoliated deciduous teeth (SHED), DPSCs, periodontal ligament stem cells (PDLSCs), bone marrow mesenchymal stem cells (BMMSCs)	Col I, Col III, matrix metalloproteinase (MMP-2), bone Sialoprotein (BSP), osteocalcin (OCN), runt-related transcription factor -2 (RUNX2), dentin sialophosphoprotein (DSPP), DMP-1	ALP activity and osteoblastic and odontoblast differentiation genes were higher in dental stem cells than BMMSCs. SHED and PDLSCs exhibited high expression of collagen, while DPSCs and PDLSCs expressed high levels of differentiation late markers. In vivo, fibrin matrix degraded and replaced by vascularized connective tissue.
Lu et al., 2015 [313]	PEG fibrinogen	In vitro	PEG fibrinogen with variable amounts of polyethylene glycol diacrylate (PEGDA)	Increase of PEGDA crosslinker allows for higher modulus but longer times of crosslinking and less swelling ratio	DPSCs	Col I, DSPP, DMP-1, OCN	Odontogenic genes expressions and mineralization were correlated to the hydrogel crosslinking degree and matrix stiffness
Jones et al., 2016 [314]	HyStem-C is a commercial hydrogel hyaluronic acid (HA)- PEGDA -gelatin	In vitro	Polyethylene glycol diacrylate with an added disulfide bond (PEGSSDA) with an added disulfide bond.	Hydrogel gelation time decreased as the PEGSSDA crosslinker concentration (*w*/*v*) increased from (0.5% to 8.0%)	DPSCs		The PEGSSDA-HA-Gn was biocompatible with human DPSCs. Cell proliferation and spreading increased considerably with adding fibronectin to PEGDA-HA-Gn hydrogels.
Feng et al., 2020 [312]	Polyethylene glycol diacrylate\sodium alginate (PEGDA/SA)	In vitro In vivo	PEGDA/SA loaded basic fibroblast growth factor (bFGF) (PEGDA/SA-bFGF)		DPSCs		Reduction of mass ratio of PEGDA/SA to 20:1 ~ 15:1 resulted in the formation of a well-organized pulp structure after implantation

**Table 4:** Comparing natural and synthetic hydrogels.

Comparing Natural and Synthetic Hydrogels						
Galler et al., 2018 [325]	- PEG - Self-assembling peptide (SAPbio) and Puramatrix™)	In vitro In vivo	- PEG was modified to be chemically cured (PEGchem) or light-cured (PEGlight), - Biomimetic hydrogels (PEGbio) modified by cell adhesion motif and MMP-2. - Self-assembling peptide (SAPbio) modified by cell adhesion motif and MMP-2.	DPSCs	TGF-β1	In vitro viability was higher in natural materials. Scaffold degradation, odontoblast-like cell differentiation, tissue formation and vascularization were higher in natural materials in vivo.
